# The Genetic Makeup of the *Drosophila* piRNA Pathway

**DOI:** 10.1016/j.molcel.2013.04.031

**Published:** 2013-06-06

**Authors:** Dominik Handler, Katharina Meixner, Manfred Pizka, Kathrin Lauss, Christopher Schmied, Franz Sebastian Gruber, Julius Brennecke

**Affiliations:** 1 Institute of Molecular Biotechnology of the Austrian Academy of Sciences (IMBA), Dr. Bohrgasse 3, 1030 Vienna, Austria

## Abstract

The piRNA (PIWI-interacting RNA) pathway is a small RNA silencing system that acts in animal gonads and protects the genome against the deleterious influence of transposons. A major bottleneck in the field is the lack of comprehensive knowledge of the factors and molecular processes that constitute this pathway. We conducted an RNAi screen in *Drosophila* and identified ∼50 genes that strongly impact the ovarian somatic piRNA pathway. Many identified genes fall into functional categories that indicate essential roles for mitochondrial metabolism, RNA export, the nuclear pore, transcription elongation, and chromatin regulation in the pathway. Follow-up studies on two factors demonstrate that components acting at distinct hierarchical levels of the pathway were identified. Finally, we define CG2183/Gasz as an essential primary piRNA biogenesis factor in somatic and germline cells. Based on the similarities between insect and vertebrate piRNA pathways, our results have far-reaching implications for the understanding of this conserved genome defense system.

## Highlights

• Systematic identification of somatic piRNA pathway factors in *Drosophila*
• Identification of functional links between piRNA biology and major cellular processes
• Characterization of CG2183/Gasz as an essential primary piRNA biogenesis factor

## Introduction

The *Drosophila melanogaster* genome contains ∼15%–20% of transposable elements (TEs) (Hoskins et al., 2002; Kaminker et al., 2002). Uncontrolled activity of TEs triggers defects in genome integrity due to DNA breaks, insertional mutagenesis, and illegitimate recombination (Levin and Moran, 2011; Slotkin and Martienssen, 2007). This is particularly evident in the germline, where TEs have evolved to be especially active but are suppressed by the piRNA pathway. The piRNA pathway is a small RNA silencing system that is defined via the involvement of PIWI family proteins bound to ∼22–31 nt PIWI-interacting RNAs (piRNAs) (Malone and Hannon, 2009; Siomi et al., 2011). Defects in the piRNA pathway allow overall development to occur, but emerging animals are sterile, presumably due to damage of their germ cell genomes (Klattenhoff et al., 2007).

Similar to other small RNA pathways, the central players in the piRNA pathway are Argonaute proteins bound to small RNAs that provide target specificity (Ghildiyal and Zamore, 2009). However, in contrast to the siRNA and the miRNA pathways, whose molecular frameworks are well described, nearly all aspects of the piRNA pathway are poorly understood. In flies, most piRNAs are derived from piRNA clusters, large loci that contain high proportions of TE sequences (Brennecke et al., 2007). piRNA cluster transcripts are believed to be exported to the cytoplasm where numerous factors participate in the processes of piRNA biogenesis and piRNA loading into PIWI proteins. Most identified piRNA biogenesis factors are enriched in distinct foci that are in proximity to the nuclear envelope (Yb-bodies in ovarian somatic cells and nuage in germline cells) (Siomi et al., 2011). Yb-bodies and nuage are tightly associated with mitochondria, and the essential piRNA biogenesis factor Zucchini is integrated into the outer mitochondrial membrane (Ipsaro et al., 2012; Nishimasu et al., 2012; Saito et al., 2010). After formation of piRNA-induced silencing complexes (pi-RISCs), the identity of the involved PIWI protein defines their respective fates. Piwi-piRNA complexes are transported into the nucleus, where they guide transcriptional silencing (Sienski et al., 2012; Wang and Elgin, 2011; Le Thomas et al., 2013; Rozhkov et al., 2013). The two other family members, Aubergine (Aub) and AGO3, remain in the cytoplasm and are engaged in the ping-pong cycle, a piRNA amplification loop that requires complementary RNAs from TEs and piRNA clusters (Brennecke et al., 2007; Gunawardane et al., 2007).

The *Drosophila* ovary contains two major cell types: germline cells derived from primordial germ cells and somatic support cells of mesodermal origin. Both cell types silence TEs by the piRNA pathway, but the respective pathways differ considerably (Senti and Brennecke, 2010; Siomi et al., 2011). While somatic support cells express only Piwi, germline cells also express Aub and AGO3. Ping-pong amplification and many factors involved in it are thus restricted to germline cells (Li et al., 2009; Malone et al., 2009).

Motivated by the gaps in our understanding of the piRNA pathway, we performed a genetic screen in the *Drosophila* ovary for factors involved in the somatic piRNA pathway. Many identified genes could be grouped into functional categories, and these indicate the identification of key factors acting at all steps of the pathway, ranging from piRNA cluster expression to cluster transcript export to piRNA biogenesis and loading and finally to piRNA-mediated silencing. Our work will therefore serve as a key resource for the genetic and mechanistic dissection of this conserved genome defense pathway.

## Results

### A Screen for Somatic piRNA Pathway Factors

**Figure 1:**
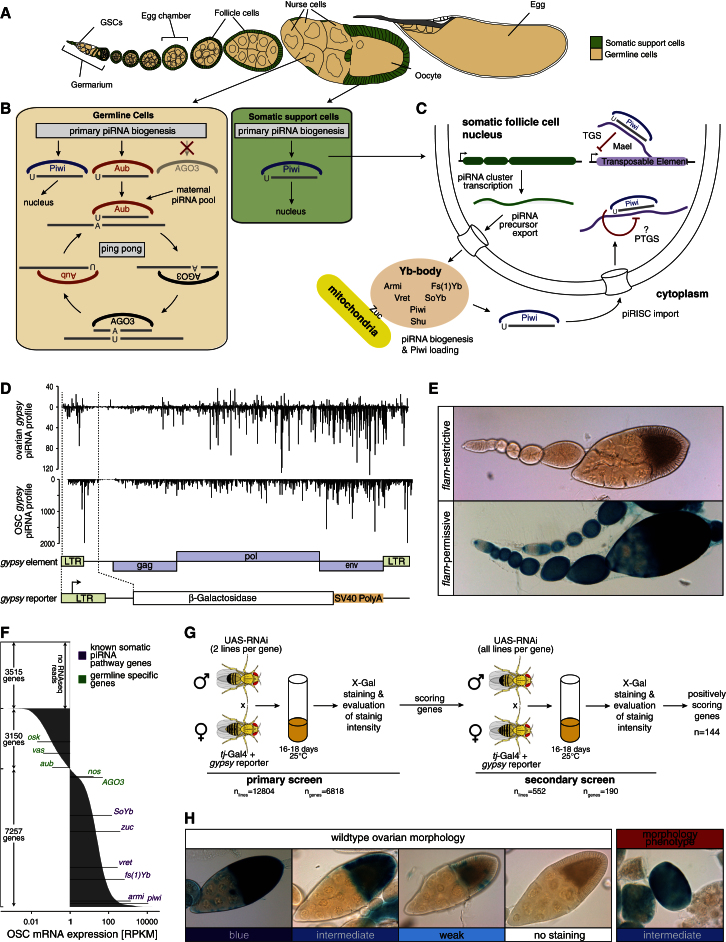
An RNAi Screen for Somatic piRNA Pathway Factors (A) Cartoon of a *Drosophila* ovariole with somatic cells in green and germline cells in beige. (B) Schematic representations of the *Drosophila* germline and somatic piRNA pathways focusing on the three PIWI family proteins and the biogenesis routes of their bound piRNAs. (C) Detailed model of the somatic piRNA pathway. Known pathway members are placed at their functional positions based on literature (TGS, transcriptional gene silencing; PTGS, posttranscriptional gene silencing). (D) Illustration of the *gypsy*-lacZ reporter. Shown are normalized profiles of ovarian and OSC piRNAs (sense up, antisense down) mapping to the *gypsy* TE and the *gypsy* sequence portion present in the reporter. (E) Shown are β-gal stainings of ovarioles as readout for the *gypsy*-lacZ reporter from *flamenco* restrictive (upper panel) or *flamenco* permissive flies (lower panel). (F) Bar chart showing expression levels (average RPKM values (log10 scale) obtained from two independent RNA-seq experiments) of all annotated *Drosophila* genes in OSCs. Several known somatic piRNA pathway factors (violet) and germline-specific control genes (green) are indicated. (G) Illustration of the screen workflow. Indicated are the numbers of tested RNAi lines and the corresponding number of genes for the primary and secondary screens. (H) Shown are β-gal stainings of representative egg chambers indicating major staining categories used for the evaluation of the screen crosses (left images, wild-type morphology; right image, distorted morphology).

The *Drosophila* ovary consists of two major cell types (somatic support cells and germline cells) that harbor different piRNA pathway versions (Figures 1A–1C). Though strong similarities exist between both pathways, it is unclear how distinct their genetic makeup is. Having an effective transgenic RNAi assay system for somatic piRNA pathway activity at hand (Olivieri et al., 2010), we screened the set of genes expressed in somatic support cells for uncharacterized piRNA pathway factors.

We took advantage of a reporter construct that expresses β-galactosidase (β-gal) under control of the *gypsy* LTR (Figures 1D and 1E; Sarot et al., 2004). The resulting RNA contains ∼700 nt of *gypsy* sequence. Many piRNAs isolated from ovaries or from cultured ovarian somatic cells (OSCs) are complementary to this region and guide efficient reporter silencing in wild-type flies (*flamenco* restrictive). Flies harboring a functional piRNA pathway but lacking the ability to produce *gypsy* complementary piRNAs (*flamenco* permissive) exhibit strong reporter desilencing. Similarly, knockdown of somatic piRNA pathway components such as Armitage (Armi), Zucchini (Zuc), or Vreteno (Vret) in a *flamenco* restrictive background results in near loss of *gypsy*-derived piRNAs and reporter desilencing (Handler et al., 2011; Olivieri et al., 2010; Saito et al., 2010; Zamparini et al., 2011). As the *gypsy*-lacZ reporter assays the endpoint of the piRNA pathway (namely piRNA-mediated silencing in *trans*), the screen should capture factors involved at all steps of the piRNA pathway (piRNA cluster biology, piRNA biogenesis, and piRNA-mediated silencing) (Figure 1C).

To filter for genes with possible functions in the somatic piRNA pathway, we determined the gene expression profile of OSCs by RNA-seq. OSCs were derived from a somatic stem cell population of the germarium and express a functional piRNA pathway (Niki et al., 2006; Saito et al., 2009). At a conservative cutoff (1 read per kilobase per 1 million sequenced reads [RPKM = 1]), OSCs express 7,257 genes, and all known somatic piRNA pathway factors were well above this cutoff (Figure 1F).

We used the collection of transgenic RNAi lines from the Vienna RNAi Center (VDRC) (Dietzl et al., 2007) and screened two lines per expressed gene. For the primary screen, this resulted in a total of 12,804 crosses for 6,818 genes (94% of expressed genes). Per cross, five F1 females were dissected and ovaries were stained for β-gal activity. Genes with at least one line exhibiting detectable reporter derepression were retested with all VDRC lines available for this gene (Figure 1G). Dissected ovaries from the secondary screen were mounted on glass slides, and β-gal activity was determined using a classification scheme with six values ranging from strong to weak (representatives shown in Figure 1H). This resulted in 144 genes where at least one knockdown line led to detectable β-gal activity (Table S1). In addition to reporter activity, we classified overall ovarian morphology as “wild-type,” “distorted,” or “rudimentary” (Figure 1H; Table S1).

### High Sensitivity, Specificity, and Reproducibility of the Screen

For an overall evaluation of the screen, we considered three questions: (1) What is the false negative rate? (2) What is the false positive rate? (3) How well does the *gypsy*-lacZ reporter mirror derepression of endogenous TEs?

**Figure 2:**
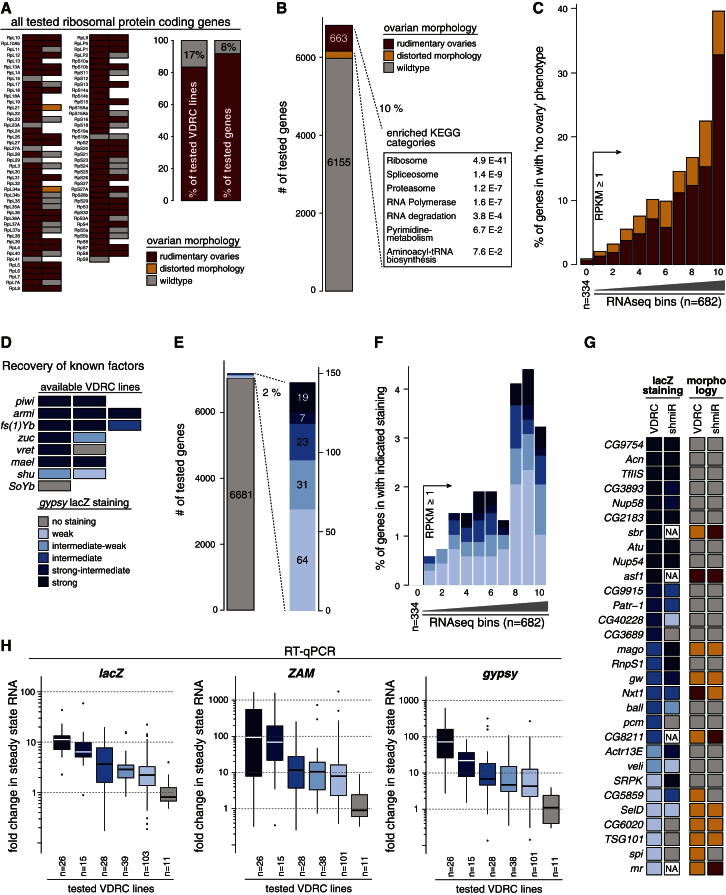
High Sensitivity, Specificity, and Reproducibility of the Screen (A) Indicated to the left is the impact on ovarian morphology observed upon knockdown of the 83 genes encoding for ribosomal proteins. The two bar charts are based on this analysis and indicate the percentage of effective VDRC lines (left) and the corresponding false negative rate (8%) at the gene level if approximately two lines per gene were tested (right). (B) Bar chart illustrating the ovarian morphology phenotype observed for all genes tested in the screen. At least one line per gene had to fall into the indicated categories. Also shown are the most enriched gene ontology (GO) categories (p values corrected for multiple testing) for the set of 663 genes that classified for the “no ovary” phenotype. (C) Indicated are the percentages of genes flagged with the “no ovary” or “distorted morphology” phenotypes when all tested genes were split into ten bins according to their expression level (gray triangle). Bins 1–10 are equally sized bins (n = 682) of all expressed genes (RPKM > 1), while bin 0 contains 334 randomly tested genes expressed below RPKM = 1. (D) Shown are the *gypsy*-lacZ staining results from the screen for available VDRC lines targeting the eight known piRNA pathway factors. (E) Bar chart summarizing the *gypsy*-lacZ staining results ranging from weak to strong for all genes tested in the screen. (F) Indicated are the percentages of genes scoring with the indicated *gypsy*-lacZ intensities when all tested genes were split into ten bins according to their expression level (gray triangle). Bins 1–10 are equally sized bins (n = 682) of all expressed genes (RPKM > 1), while bin 0 contains 334 randomly tested genes expressed below RPKM = 1. (G) Compared are *gypsy*-lacZ intensities as well as the morphology phenotypes for 30 screen-positive genes tested with shRNA lines or VDRC lines (NA, not analyzable due to a “no ovary” phenotype). (H) Shown are box plots displaying the fold changes in steady-state RNA levels (based on RT-qPCR) of *lacZ*, *ZAM*, and *gypsy* normalized to control knockdowns. Tested were all RNAi lines (numbers given at the bottom) falling into the five staining categories (color coded) and 11 control lines (gray). Box plots show median (line), 25th–75th percentile (box) ± 1.5 interquartile range; circles represent outliers.

The number of known genes acting in the somatic piRNA pathway is limited (n = 8) and not well suited for a false negative estimation. To determine the efficacy of VDRC RNAi lines, we evaluated the ovarian morphology resulting from knockdowns of genes encoding for ribosomal proteins (n = 83). Defective ribosome biogenesis results in a “rudimentary ovary” phenotype, and based on this, ∼83% of VDRC lines elicit efficient target knockdown (Figure 2A). As we typically tested two independent lines per gene, this resulted in a ∼92% positive rate at the gene level. Similar results were obtained when other housekeeping categories were analyzed. Consequently, the set of 663 genes resulting in rudimentary ovaries upon knockdown was highly enriched in basic cellular processes (Figure 2B). We observed a correlation between the “rudimentary ovary” phenotype and gene expression levels, with knockdowns of nearly 40% of the 700 most highly expressed genes preventing ovary development (Figure 2C). We conclude that transgenic RNAi is potent in knocking down genes with very high mRNA levels. As we also tested 358 genes below the RPKM cutoff of 1, we can estimate that off-target effects are not prevalent in this screen, as only 0.8% of lines targeting these “nonexpressed” genes resulted in altered ovarian morphology.

Eight genes have been assigned to the somatic piRNA pathway, and with the exception of the Tdrd12 member SoYb (the available VDRC line is nonfunctional), all of these were identified among the top scoring candidates (Figure 2D) (Haase et al., 2010; Handler et al., 2011; Malone et al., 2009; Olivieri et al., 2010, 2012; Preall et al., 2012; Qi et al., 2011; Saito et al., 2010; Sienski et al., 2012; Zamparini et al., 2011). We conclude that the screen identified most somatic piRNA pathway factors whose knockdown results in analyzable ovaries.

The false positive rate depends on the extent of the *gypsy-*lacZ sensor being influenced only by the piRNA pathway and also on the extent of screen artifacts such as off-targets. Remarkably, only 2% (144 genes) of all tested genes showed any β-gal activity, and only 0.7% (49 genes) showed activity in the range of known pathway members (Figure 2E). Also, for the positive genes, we observed a correlation with expression levels (Figure 2F). This was, however, less pronounced compared to the morphology results (Figure 2C). Importantly, very few of the weakly expressed genes and none of the 358 tested nonexpressed genes derepressed the reporter upon knockdown.

To more directly assess the specificity of the screen, we performed two tests. First, we generated 30 shRNA lines (Ni et al., 2011) to reassess the screening results with independent knockdowns (Figure 2G). Out of 26 lines that could be analyzed (four lines resulted in “rudimentary ovaries”), 21 (>80%) confirmed the screen result. Considering that not all shRNA lines will be functional, RNAi off-target effects can be considered to be very low in the screen. We then asked whether the β-gal results correlate with endogenous TE derepression. We performed RT-qPCR experiments on ovarian RNA after knocking down each positively scoring gene in somatic follicle cells. We measured steady-state RNA levels of β-gal as well as of the endogenous retro-elements *ZAM* and *gypsy*, which are under control by the somatic piRNA pathway. In all cases, the classification of β-gal reporter activity correlated highly with the measured RNA levels (Figure 2H; Table S2). Taken together, our analyses indicate a very high sensitivity and specificity of the screen.

### Classification of Germline-Specific, Soma-Specific, and Common piRNA Factors

The existence of distinct piRNA pathways in the ovarian soma and germline poses the question about their genetic similarities. We therefore tested the identified candidates from the soma screen for their involvement in germline TE repression.

**Figure 3:**
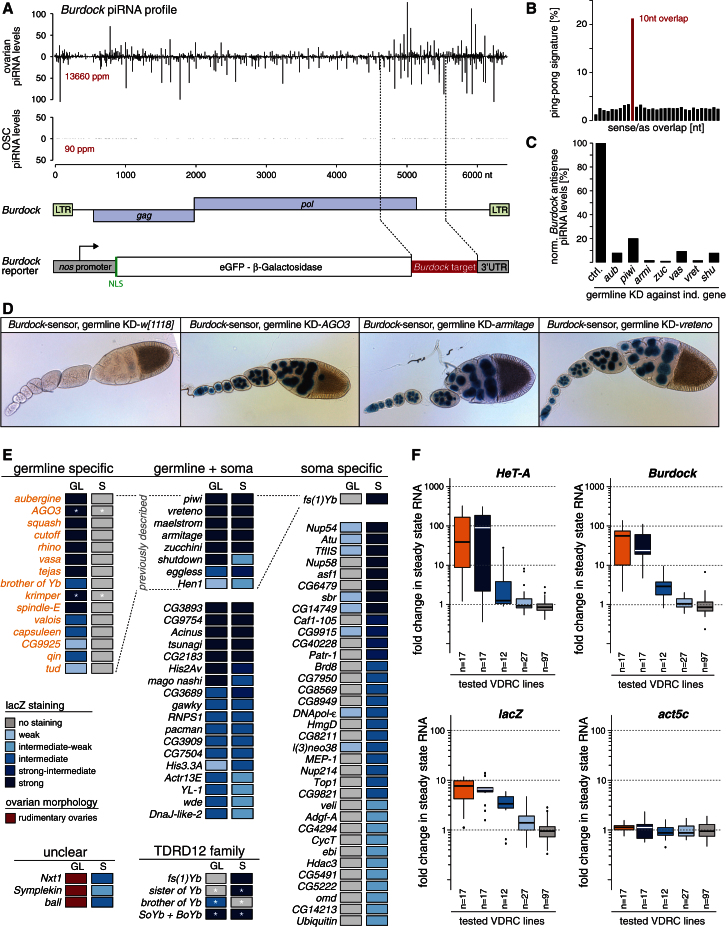
Genetic Classification of Germline-Specific, Soma-Specific, and Common piRNA Factors (A) Illustration of the *Burdock*-lacZ reporter. Shown are normalized profiles of ovarian and OSC piRNAs (sense up, antisense down) mapping to the *Burdock* element and the *Burdock* sequence portion present in the 3′ UTR of the reporter, which expresses β-gal under control of the germline-specific *nanos* promoter. (B) Bar diagram displaying the sense/antisense overlap patterns of the ovarian piRNA population mapping to the *Burdock* element. The red bar at 10 nt indicates a significant ping-pong signature. (C) Bar plot illustrating normalized piRNA levels (%) antisense to the *Burdock* TE in ovaries from indicated germline-specific knockdowns (MTD × shRNA) compared to control levels. (D) Shown are β-gal stainings of egg chambers expressing the *Burdock*-lacZ reporter and germline knockdowns (KD) for the indicated genes (*w[1118]* serves as negative control). (E) Listed are all genes known to be specific for the germline piRNA pathway (orange set) and all genes scoring in the somatic screen (intermediate-weak or stronger; no mitochondrial genes). Based on the staining intensities observed with the *Burdock*-lacZ reporter (GL/left columns) or the *gypsy*-lacZ reporter (S/right columns), genes were grouped into germline-specific, common, and soma-specific classes. All genes previously linked to the piRNA pathway are marked as “previously described.” Genes not interpretable in the germline test are shown in the lower left panel. Asterisks indicate genes tested with an shRNA line because no VDRC line was available (*AGO3*, *krimper*) or the VDRC line is not functional (*SoYb*). (F) Box plots showing fold deregulations of *HeT-A*, *Burdock*, *lacZ*, and *act5c* RNA levels upon knockdown of all screen-scoring VDRC lines. Lines were grouped into three staining categories (blue) based on their effect on the *Burdock*-lacZ reporter. Germline-specific factors (orange) are represented as a separate group.

We depleted screen candidates in the germline by transgenic RNAi based on the *nanos* GAL4 driver coupled to UAS-Dcr2 and VDRC lines (Handler et al., 2011; Wang and Elgin, 2011). To monitor piRNA pathway integrity in germline cells, we generated a TE repression sensor. This reporter expresses nuclear β-gal under control of the *nanos* promoter and harbors a target sequence for *Burdock* piRNAs in its 3′ UTR (Figure 3A). The retro-element *Burdock* is a prototypic germline TE, as ovarian piRNAs exhibit a pronounced ping-pong signature and are specifically found in germline cells but not in OSCs (Figures 3A and 3B). *Burdock* piRNA levels are sensitive to loss of various germline piRNA pathway factors (Figure 3C), and knockdown of pathway factors as diverse as AGO3, Vreteno, and Armitage strongly derepressed the sensor (Figure 3D). This suggested that the *Burdock* sensor is a valid tool to monitor germline piRNA pathway integrity. Knockdown of any known major germline piRNA pathway factor led to robust sensor activation (Figure 3E). Even knockdown of candidates with moderate impacts on TE silencing (Tudor, Hen1, CG9925) (Handler et al., 2011; Horwich et al., 2007; Nishida et al., 2009) derepressed the sensor, albeit to lesser extents (Figure 3E). Of note, every tested VDRC line targeting a validated germline piRNA pathway factor was highly efficient in this system. We conclude that the germline knockdown system is very robust, allowing meaningful conclusions about the tissue specificity of identified piRNA pathway factors.

Based on the *Burdock*-lacZ sensor, 26 factors scoring at least weak-intermediate in the screen also scored in the germline (Figure 3E). Remarkably, ∼30 genes scored specifically in the somatic piRNA pathway only. Among these were some of the strongest scoring screen candidates, and several were validated via independent shRNA lines (Figure 2G). Our results strengthen large similarities between the soma and the germline piRNA pathways but also pinpoint significant differences.

To substantiate the validity of *Burdock*-lacZ as an accurate proxy for germline piRNA pathway integrity, we measured steady-state RNA levels of the two germline-specific TEs, *HeT-A* and *Burdock*. We observed strong correlations with the *Burdock*-lacZ results (Figure 3F; Table S3). Knockdown of candidates strongly scoring in the germline derepressed endogenous TEs to an extent that was highly similar to knockdowns of established germline piRNA pathway factors. On the other hand, no significant changes in endogenous TE levels were seen upon knockdown of factors that scored in the soma but not in the germline. The housekeeping gene *Actin 5C* served as control.

Taken together, our results define the set of common factors acting in the somatic and germline piRNA pathways and provide genetic entry points to dissect the differences between the two pathways. Reaching a similar status for the class of “germline-specific” factors would require a targeted germline screen as presented in the accompanying manuscript by Hannon and colleagues (Czech et al., 2013).

### Identification of Key Processes Involved in the Somatic piRNA Pathway

**Figure 4:**
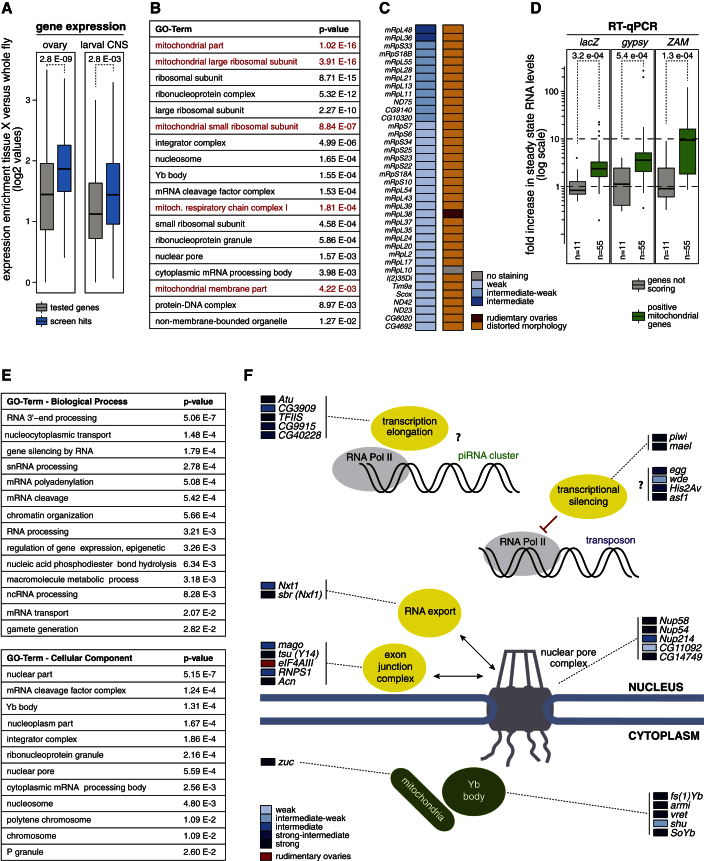
Key Processes and Factors Involved in the Somatic piRNA Pathway (A) Box plots showing the fold enrichment of gene expression (based on FlyAtlas) in ovaries or larval CNS versus whole flies for all tested genes (gray) and the set of positive screen hits (blue); p values were determined by Wilcoxon signed rank test. (B) Shown are the most significantly enriched GO terms among the 144 scoring genes in the screen (p values corrected for multiple testing). Mitochondria-related terms are in red. (C) Listed are all genes with annotated mitochondrial function and their respective *gypsy*-lacZ staining and ovarian morphology phenotypes observed in the somatic screen. (D) Box plots showing fold increases in *lacZ*, *gypsy*, and *ZAM* steady-state RNA levels for the set of mitochondrial gene knockdowns (green box) compared to nonscoring genes (gray box); p values were determined by Wilcoxon signed rank test. Box plots are defined in Figure 2H. (E) Significantly enriched GO terms among screen hits without mitochondria associated genes (p values corrected for multiple testing). (F) Cartoon depicting functional groups of genes identified in the screen. Factors were grouped and placed into nucleus or cytoplasm based on their annotated functions or by identification of orthologous genes with annotated functions. Genes involved in the various processes are indicated together with the *gypsy*-lacZ staining results.

A comparison of all 144 screen hits to the set of 6,818 tested genes in respect to tissue gene expression profiles (FlyAtlas, http://www.flyatlas.org; Chintapalli et al., 2007) indicated that the expression of scoring genes was higher in ovaries and to a lesser extent in the larval CNS compared to whole flies (Figure 4A). These results are in agreement with the piRNA pathway acting specifically or preferentially in gonads.

The set of 144 candidate piRNA pathway genes was highly enriched for several functional gene ontology (GO) terms (Figure 4B). Surprisingly, a number of top categories were associated with mitochondrial metabolism. Indeed, 23% of all screen hits (38 genes) could be annotated as mitochondrial proteins (Figure 4C). All of these derepressed the *gypsy*-lacZ sensor only moderately and impacted ovarian morphology to varying degrees. Based on RT-qPCR results, knockdown of identified mitochondrial factors increased the expression level of endogenous TEs moderately but significantly (Figure 4D). As many other factors whose knockdown impaired oogenesis did not result in sensor activation, we conclude that mitochondrial integrity is important for full piRNA pathway activity. Interestingly, the essential piRNA biogenesis factor Zuc is an integral protein of the outer mitochondrial membrane, and piRNA biogenesis is proposed to occur in close proximity to mitochondria. We note that knockdown of mitochondrial genes in the germline generally did not result in measurable *Burdock*-lacZ sensor derepression, suggesting that the somatic piRNA pathway is more sensitive to mitochondrial malfunction.

We repeated the GO analysis after subtracting annotated mitochondrial proteins. Many of the significantly enriched categories (Figure 4E) were linked to RNA biology, nucleo-cytoplasmic transport, or chromatin biology. In combination with manual annotations, we propose the involvement of several functionally or genetically related gene groups in the piRNA pathway (Figure 4F). In the cytoplasm, several factors required for piRNA biogenesis (Yb, Armi, Vret, Shu) are enriched in Yb-bodies, which are in direct vicinity of mitochondria (Szakmary et al., 2009). Three factors with relationships to processing bodies (Gawky, Pacman, Patr-1) suggest a connection between the piRNA pathway and P-bodies. Notably, several piRNA pathway factors colocalize with P-bodies in so-called piP-bodies in mouse male germ cells (Aravin et al., 2009), and Yb-bodies have been observed in proximity to P-bodies in *Drosophila* follicle cells (Olivieri et al., 2010).

More intriguingly, we identified several factors with a nuclear function or that act at the interface between nucleus and cytoplasm. In each category, at least one factor was among the strongest-scoring candidates, and independent shRNA lines validated several factors in each category.

The two core exon junction complex (EJC) (Tange et al., 2004) factors Mago and Tsunagi/Y14 as well as the EJC accessory factors RnpS1 and Acinus scored strongly in the assay. The third core factor, eIF4AIII, could not be analyzed due to a “rudimentary ovary” phenotype. All identified EJC factors also classified as essential pathway factors in the germline (Figure 3E), strongly implicating the EJC in piRNA pathway biology.

The central RNA export factors small bristles (Nxf1) and Nxt1 strongly derepressed the sensor. These factors are implicated in general mRNA export, and ovarian morphology was distorted in the respective knockdowns. Nevertheless, the extent of sensor and TE derepression upon knockdown of these factors hints at a direct involvement of this protein complex, potentially at the level of piRNA cluster transcript export. Interestingly, Nxf2 and Nxf3 are specifically expressed in ovaries (but not in OSCs), and their knockdown in the germline results in TE silencing defects (unpublished observations), strongly substantiating the central importance of RNA export processes in the piRNA pathway.

Four nuclear pore complex factors (Nup54, Nup58, CG11092, Nup214) and one accessory factor (CG14749) scored in the screen. The two FG repeat proteins Nup54 and Nup58 were among the strongest-scoring candidates, and given that ovarian morphology was normal in the respective knockdowns, we postulate that an intricate and specific connection exists between the nuclear pore complex and piRNA biology. All of these factors appear to be specifically involved in the soma piRNA pathway (Figure 3E). Our finding might relate to a recent study in germline cells that found piRNA cluster loci specifically at nuclear pores to allow for specific and/or efficient coupling of piRNA cluster transcription/export to the perinuclear piRNA biogenesis machinery (Zhang et al., 2012).

Several strongly scoring factors are annotated to be involved in aspects of RNA Polymerase II biology. For example, the identified factors Atu, CG3909, CG9915, CG40228, and TfIIS are either annotated Paf1 complex members or are linked to TfIIS, a key factor required to rescue backtracked RNA Pol II (Sims et al., 2004). These proteins might be involved in Piwi-mediated transcriptional silencing. However, the group of factors with links to Pol II did not score in the germline. As Piwi-mediated TE silencing is unlikely to differ between somatic and germline cells, we favor the hypothesis that factors ensuring efficient Pol II activity are important for aspects of piRNA cluster biology. *flamenco*, for example, is a locus that spans at least 180 kb of repetitive sequence, and all evidence suggests that it is transcribed from a single promoter. Productive transcription through such a large locus might well require factors that reactivate backtracked polymerase. The soma-specific requirement of these factors suggests that transcription of germline piRNA clusters differs from that of *flamenco*. This might relate to their bidirectional transcription and the specific requirement for Rhino and Cutoff for their transcription/processing (Klattenhoff et al., 2009; Pane et al., 2011).

Piwi-mediated silencing of TEs occurs predominantly at the transcriptional level (Sienski et al., 2012; Wang and Elgin, 2011; Le Thomas et al., 2013; Rozhkov et al., 2013). Besides Piwi and Maelstrom, we identified several factors with chromatin-associated functions that might participate in this process. Among these are the histone variant His2Av, the histone chaperone Asf1, the H3K9 methyltransferase Eggless and its cofactor Windei, as well as others. Of note, most of these factors also scored in the germline (Figure 3E).

### Uncharacterized Factors in piRNA Biogenesis and Piwi-Mediated Silencing

**Figure 5:**
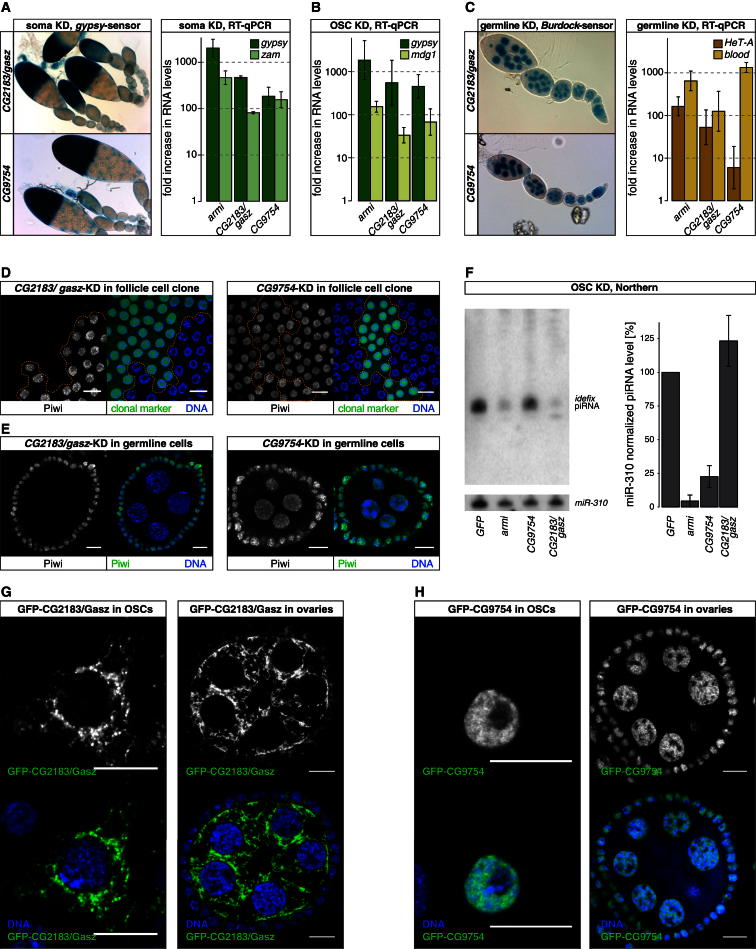
CG2183/Gasz and CG9754 Are Essential for the Somatic piRNA Pathway (A) Left panels show β-gal stainings of ovarioles as readout for *gypsy*-lacZ silencing upon soma knockdown of *CG2183*/*gasz* or *CG9754*. The bar diagram depicts fold increases in RNA levels of indicated TEs in ovaries with soma knockdown of *armi*, *CG2183*/*gasz*, or *CG9754* (averages of three biological replicates; error bars, SD; normalized to control knockdown). (B) Displayed are fold increases in RNA levels of indicated TEs in OSCs upon siRNA-mediated knockdown of *armi*, *CG2183*/*gasz*, or *CG9754* siRNA in OSCs (averages of three biological replicates; error bars, SD; normalized to control knockdown). (C) Left panels show β-gal stainings of ovarioles as readout for *Burdock*-lacZ silencing upon germline knockdown of *CG2183*/*gasz* or *CG9754*. The bar diagram depicts fold increases in RNA levels of indicated TEs in ovaries with germline knockdown of *armi*, *CG2183*/*gasz*, or *CG9754* (averages of three biological replicates; error bars, SD; normalized to control knockdown). (D) Confocal sections (scale bars, 10 μm) through the follicular epithelium of egg chambers stained for Piwi (monochrome panel), DNA (blue), and the clonal marker (green). Cells within the clone (dashed lines mark clone boundaries in the monochrome panels) express dsRNAs against *CG2183*/*gasz* (left panel) or *CG9754* (right panel). (E) Confocal sections (scale bars, 10 μm) through egg chambers stained for Piwi (monochrome panel and green) and DNA (blue). Knockdown of *CG2183*/*gasz* (left) and *CG9754* (right) was specifically activated in germline cells. (F) Northern blot analysis of piRNA levels in OSC total RNA upon siRNA-mediated knockdowns of *GFP* (control), *armi*, *CG9754*, or *CG2183*/*gasz*. One representative blot probed for *idefix* piRNA (top) and then reprobed for *miR-310* (bottom) is shown. The bar diagram indicates quantified results (normalized to *miR-310*) based on three independent experiments (error bars, SD.). (G) Confocal sections (scale bars, 10 μm) of OSCs (left panels) or egg chambers (right panels) expressing GFP-CG2183/Gasz stained for DNA (blue). Monochrome panels show the GFP signal separately. (H) Confocal sections (scale bars, 10 μm) of OSCs (left panels) or egg chambers (right panels) expressing GFP-CG9754 stained for DNA (blue). Monochrome panels show the GFP signal separately. See also Figure S1.

Many genes identified in the screen do not belong to a functional category. Among these, we chose *CG2183* and *CG9754*, two uncharacterized genes, for follow-up studies. Knockdown of either gene in somatic follicle sells resulted in strong *gypsy*-lacZ sensor derepression (Figure 5A, left) as well as in derepression of the endogenous TEs *gypsy* and *ZAM* (Figure 5A, right). The extent of TE derepression was comparable to knockdowns of Armi, an essential piRNA biogenesis factor. We confirmed these findings with independent shRNA transgenes (Figure 2G, not shown). In OSCs, siRNA-mediated knockdown of *CG2183* or *CG9754* led to strong increases in RNA levels of *gypsy* and *mdg1*, both of which are piRNA targets in this cell line (Figure 5B). Both genes classified also as essential piRNA pathway factors in germline cells. We based this on the *Burdock*-lacZ sensor results and RT-qPCR analyses of the germline-repressed TEs *HeT-A* and *blood* (Figure 5C). Overall, knockdown of these two genes resulted in highly similar defects in TE repression.

However, when analyzing the central effector protein Piwi as well as piRNA levels, clear differences between the two factors emerged. While depletion of CG2183 in follicle cells or germline cells resulted in a near loss of Piwi, depletion of CG9754 had no detectable impact on Piwi levels or localization (Figures 5D and 5E). Multiple piRNA biogenesis pathways feed into the three PIWI family proteins in ovaries, complicating analyses of piRNA biogenesis. We therefore probed for an involvement of CG2183 or CG9754 in piRNA biogenesis in OSCs, which exhibit only primary piRNA biogenesis. Judged by northern analysis, levels of mature piRNAs were reduced to ∼25% upon CG2183 depletion but were unchanged upon CG9754 depletion (Figure 5F). We sequenced small RNA populations from *CG2183* versus *GFP* siRNA-treated OSCs. This confirmed a ∼4-fold reduction of piRNAs originating from annotated TEs, as well as those originating uniquely from the *flamenco* cluster or from the *traffic jam* 3′ UTR (Figure S1). Based on these results, CG2183 is a likely primary piRNA biogenesis factor.

CG9754, on the other hand, is to our knowledge most probably an uncharacterized factor essential for the Piwi-mediated silencing process. Cells depleted for CG9754 exhibited nuclear Piwi loaded with piRNAs, yet the Piwi-RISC was not able to elicit efficient TE silencing. In support for this, GFP-CG9754 localized to the nucleus (where Piwi-mediated silencing occurs) while GFP-CG2183 localized to the cytoplasm (where piRNA biogenesis occurs) both in OSCs as well as in ovaries (Figures 5G and 5H). Both GFP transgenes include the respective endogenous regulatory regions. The GFP fusion proteins were clearly detectable in ovarian somatic and germline cells (GFP-CG2183 was more abundant in germline cells) in support of both proteins being essential piRNA pathway factors in both pathways.

### CG2183 Colocalizes with Zucchini on Mitochondria and Is an Essential piRNA Biogenesis Factor

**Figure 6:**
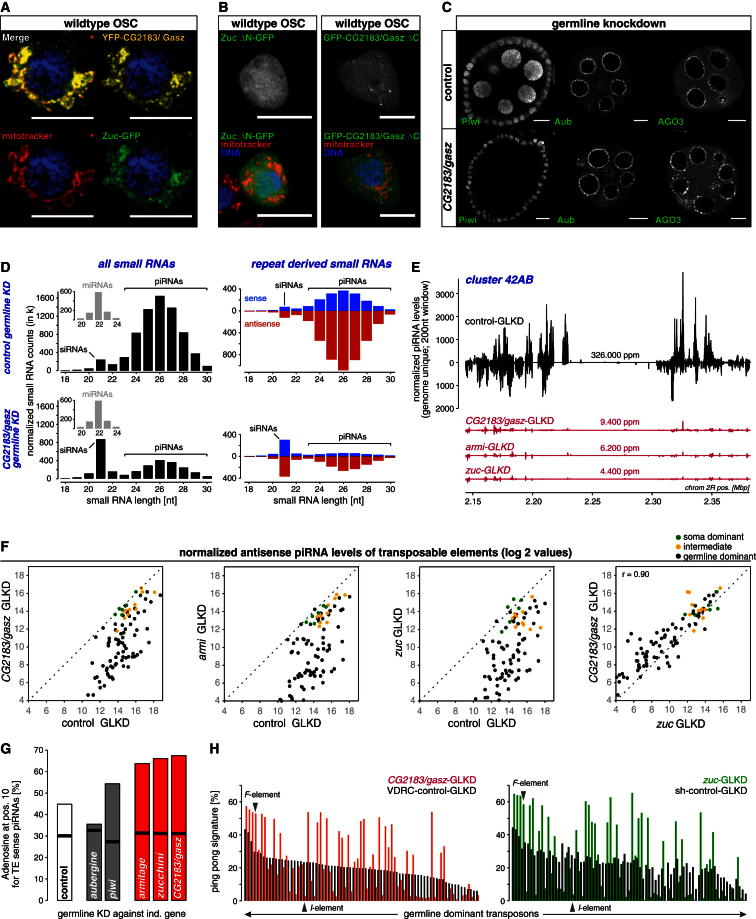
CG2183/Gasz Is an Uncharacterized piRNA Biogenesis Factor (A) Confocal sections (scale bars, 10 μm) of OSCs transfected with YFP-CG2183/Gasz (yellow) and Zuc-GFP (green) expression constructs and stained for mitochondria (MitoTracker; red) and DNA (blue). (B) Confocal sections (scale bars, 10 μm) of OSCs transfected with Zuc-GFP and GFP-CG2183/Gasz expression constructs (green) lacking the respective transmembrane domains and stained for mitochondria (MitoTracker; red) and DNA (blue). (C) Confocal sections (scale bars, 10 μm) through egg chambers stained for Piwi, Aub, or AGO3. Control knockdown or *CG2183*/*gasz* knockdown was specifically activated in germline cells. (D) Left panels show length profiles of normalized small RNA populations from ovaries with control (upper) or *CG2183*/*gasz* (lower) germline knockdown split into miRNAs (small insets) and remaining RNAs (siRNA and piRNA populations indicated). Right panels show respective length profiles of repeat-derived small RNAs only (red, antisense; blue, sense). (E) Normalized piRNA profiles (genome unique; sense up, antisense down) from ovaries with indicated germline knockdowns mapping to cluster *42AB*. (F) Scatter plots showing normalized antisense piRNA levels (log2 values) mapping to soma-dominant (green), intermediate (yellow), or germline-dominant (black) TEs from ovaries with indicated germline knockdowns (Pearson correlation [r] based on all TEs). (G) Bar chart displaying the adenosine content at position 10 for sense piRNAs mapping to TEs isolated from ovaries with indicated germline knockdowns. Black lines indicate the expected level based on the average 10A content at positions 2–9 and 11–23. (H) Shown are ping-pong signatures of germline-dominant TEs based on piRNAs from ovaries with indicated germline knockdowns. TEs were ordered according to their ping-pong signature in the VDRC control library. See also Figure S2.

The subcellular localization of GFP-CG2183 was highly reminiscent of mitochondrial patterns and of the reported Zuc localization (Saito et al., 2010). In fact, ectopically expressed Zuc-GFP and YFP-CG2183 in OSCs exhibited an indistinguishable pattern that nearly fully overlapped with mitochondria (Figure 6A). Also, in ovaries, Zuc-GFP and GFP-CG2183 colocalized significantly with mitochondria (Figure S2). When analyzing the Zuc and CG2183 polypeptides with TMHMM (Sonnhammer et al., 1998), a prediction tool for transmembrane (TM) helices, a significant TM helix was predicted for the Zuc N terminus (Saito et al., 2010) and the CG2183 C terminus (Figure S2). Deletion of the respective TM helices resulted in a uniform distribution of both proteins in OSCs (Figure 6B). This indicated that CG2183 is a transmembrane protein like Zuc, most probably of the outer mitochondrial membrane.

Our results suggested a link between CG2183 and Zuc. Indeed, knockdown of CG2183 in the germline resulted in a loss of Piwi, no change in Aub localization, and a mild delocalization of AGO3 from nuage, a pattern indistinguishable from the effects reported upon Zuc knockdown (Figure 6C) (Olivieri et al., 2010). To extend this analysis, we sequenced small RNAs from ovaries with CG2183 germline knockdown and compared piRNA populations to control as well as to Zuc or Armi knockdowns (Figure 6E). If compared to the respective control libraries (normalized to miRNA levels), piRNA populations (23–30 nt small RNAs) were reduced to 25%, 20%, or 23% upon knockdown of CG2183, Zuc, or Armi, respectively. piRNA populations mapping to the major germline piRNA cluster *42AB* collapsed nearly entirely in all three knockdowns (Figure 6E), while piRNAs derived from the soma-specific *flamenco* cluster were essentially unchanged, in agreement with the knockdown being germline specific. The similarities in piRNA populations from CG2183, Zuc, or Armi knockdowns extended to TE piRNA profiles. Highly similar patterns of piRNA losses were seen in all three cases (Figure 6F). Zuc and Armi are required for primary piRNA biogenesis but not for ping-pong-mediated piRNA biogenesis per se and have been classified as type I piRNA biogenesis factors (Olivieri et al., 2012). Loss of either factor has a highly stereotypical impact on piRNA populations. While piRNAs mapping to most TEs are severely reduced, a handful of TEs exhibit nearly unchanged piRNA levels, and these appear to be almost exclusively produced via ping-pong based on a strong increase in the 10 nt overlap of sense/antisense piRNAs. A detailed ping-pong analysis indicated that CG2183 classifies as a third type I biogenesis factor. Adenosine levels at position 10 of sense piRNAs (an indicator of ping-pong piRNA biogenesis) were increased to almost identical levels in all three knockdowns (Figure 6G). Similarly, ping-pong signatures were elevated for the same subset of TEs in CG2183 or Zuc knockdowns (Figure 6H). The two prototypical TEs *F*-element (increased ping-pong) and *I*-element (collapsed piRNA profile) exemplify the differential impact of loss of type I biogenesis factors on piRNA populations (Figure S2).

Taken together, CG2183 classifies as a type I piRNA biogenesis factor like Zuc and Armi, and its subcellular localization on mitochondria hints at an intricate connection to the endonuclease Zuc.

### *CG2183* Encodes *Drosophila* Gasz and Recruits Armitage to Mitochondria

Protein BLAST searches identified orthologs of CG2183 in mammals, and intriguingly, the mouse ortholog GASZ (*g*erm cell protein with *a*nkyrin repeats, *s*terile alpha motif, and leucine *z*ipper) has been linked to piRNA biogenesis (Ma et al., 2009). Based on the overall sequence identity of 26%, a similar domain organization (five ankyrin repeats and a sterile alpha motif), a predicted C-terminal transmembrane domain in both proteins, and a demonstrated colocalization of GASZ with mitochondria, we termed CG2183 *Drosophila* Gasz.

**Figure 7:**
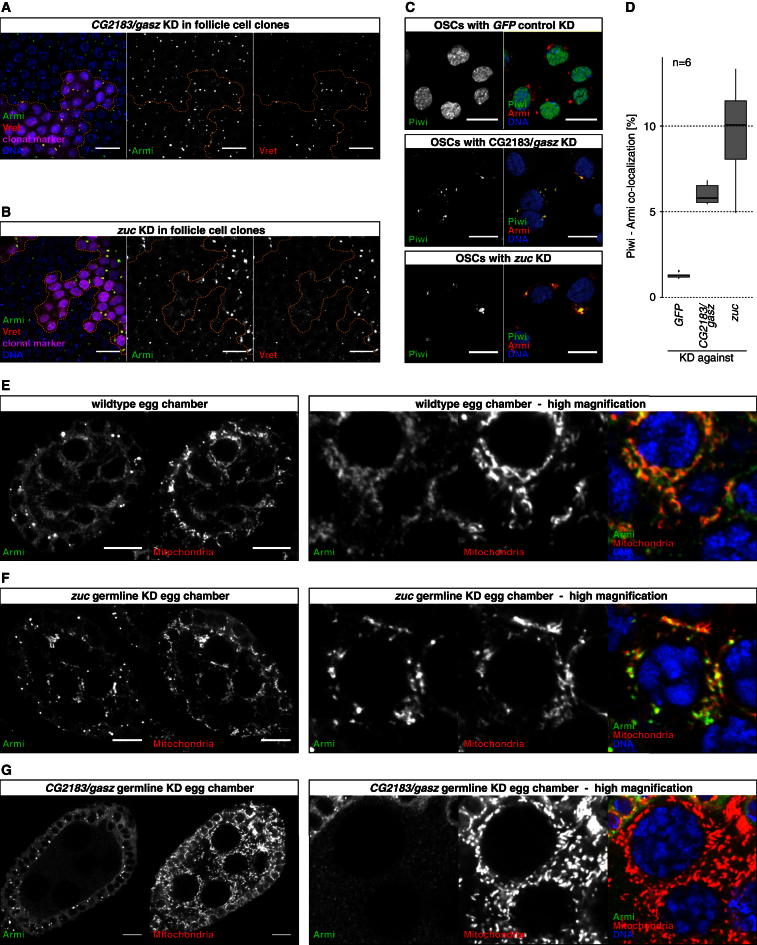
CG2183/Gasz Recruits Armitage to Mitochondria (A and B) Confocal sections (scale bars, 10 μm) through the follicular epithelium of egg chambers stained for Armi (green), Vret (red), and DNA (blue). Knockdown of *CG2183*/*gasz* (A) or *zuc* (B) was clonally induced (clonal marker in magenta; clone borders marked by dashed line). (C) Confocal sections (scale bars, 10 μm) of OSCs with indicated siRNA-mediated knockdowns stained for Piwi (green), Armi (red), and DNA (blue). (D) Quantification of Piwi-Armi colocalization based on (C). The fraction of Piwi-positive pixels colocalizing with Armi-positive pixels is indicated. Box plots are based on six quantified images per knockdown with ∼30 cells each. (E–G) Confocal sections (scale bars, 10 μm) through egg chambers with indicated genotype stained for Armi (green), mitochondria (red), and DNA (blue). Overview panels are shown to the left and high magnification images to the right. Colocalization of Armi and mitochondria in the merge panels appears yellow. See also Figures S3–S5.

Ankyrin repeats and sterile alpha motifs often mediate protein-protein interactions. Given the localization of Gasz and the endonuclease Zuc on mitochondria, we speculated that Gasz might serve as an adaptor to recruit other biogenesis factors to mitochondria. We first verified that Gasz and Zuc localize to mitochondria independent of each other, as would be predicted for two proteins harboring TM domains (Figure S3). We then tested whether any of the known piRNA biogenesis factors showed altered subcellular localization upon loss of Gasz. In follicle cells as well as in cultured OSCs, the biogenesis factors Yb, Armi, Vret, and Shu are cytoplasmic and are enriched in perinuclear Yb-bodies that depend on the RNA helicase Yb. Yb-bodies are in close association with mitochondria and are hypothesized to be the sites of primary piRNA biogenesis. We generated clones of cells expressing either *gasz* or *zuc* dsRNA hairpins in the follicular epithelium and stained for known biogenesis factors (Figures 7A and 7B). As reported previously, cells with Zuc knockdowns exhibited significantly enlarged Yb-bodies (evidenced by the accumulation of Armi and Vret). This likely results from a clustering of mitochondria into one or two perinuclear spheres per cell (Figure 7B) (Olivieri et al., 2010, 2012). Cells depleted for Gasz showed no defects in Yb-body localization of Yb, Armi, or Vret. Vret levels were elevated in Yb-bodies, but Armi levels were not, indicating no general enlargement of Yb-bodies and presumably also no clustering of mitochondria (Figure 7A). The enlarged Yb-bodies in Zuc mutant cells also stain positive for Piwi, indicating that presumably unloaded Piwi is recruited to Yb-bodies/mitochondria and cannot be released in the absence of Zuc (Olivieri et al., 2010; Saito et al., 2010). Interestingly, loss of Gasz also resulted in Piwi accumulation in Yb-bodies, albeit to a lesser degree, probably because of no apparent mitochondrial clustering (Figures 7C and 7D; Figure S4). These results once more highlighted the similarities between Zuc and Gasz, yet they did not provide any clues toward the molecular function of Gasz.

*Drosophila* somatic ovarian cells are special, as here piRNA biogenesis depends on the soma-specific RNA Helicase Yb, the defining factor of Yb-bodies (Szakmary et al., 2009). We hypothesized that localization of biogenesis factors to Yb-bodies might not be the relevant assay to test whether Gasz serves as an adaptor to recruit biogenesis factor(s) to the mitochondrial surface. We therefore analyzed the localization of piRNA biogenesis factors in germline cells (which lack Yb-bodies but do perform primary piRNA biogenesis) upon depletion of Zuc or Gasz. Striking differences were observed for the RNA Helicase Armi. In wild-type nurse cells, Armi localized to the cytoplasm and was enriched in diffuse “clouds,” which colocalized with mitochondria (Figure 7E). Remarkably, Armi accumulated even more in areas of mitochondrial clusters upon knockdown of Zuc but was evenly dispersed and lacked mitochondrial colocalization in cells depleted for Gasz (Figures 7F and 7G). Given that Piwi accumulated around mitochondrial clusters in Zuc-depleted but not in Gasz-depleted cells (Figure S5), we propose that Gasz serves as the adaptor protein that recruits an Armi-Piwi complex to mitochondria, where the endonucleolytic activity of Zuc generates the 5′ end of pre-piRNAs that will be loaded into Piwi.

Our results define a second mitochondrial transmembrane protein in primary piRNA biogenesis and provide evidence that Gasz couples biochemical reactions occurring at the mitochondrial surface (e.g., Zuc-mediated cleavage) with the upstream sorting of piRNA precursors.

## Discussion

The mechanistic understanding of the piRNA pathway is very limited, and systematic studies are hampered due to its gonad-restricted activity. Therefore, our in vivo RNAi screen provides an invaluable entry point into the dissection of this silencing system. In the following, we critically assess two key aspects that are relevant to this work.

### Specificity and Validity of the Screen Results

Transgenic RNAi screens based on the expression of long dsRNA hairpins must be rigorously evaluated in terms of their false negative and false positive rates. The false negative rate is influenced by the availability of VDRC lines (94% for this screen), by the number of genes that cannot be evaluated due to overall defects in ovarian development (10% for this screen), and by the knockdown efficiency of the VDRC lines toward their respective target genes (estimated to be ∼90% if two lines per gene were tested). Based on this, we conservatively estimate that our screen captured up to 80% of the key factors involved in the somatic piRNA pathway. We point out that the *gypsy*-lacZ sensor faithfully recapitulates defects at all levels of the piRNA pathway, such as *flamenco* mutations, piRNA biogenesis defects, or defects in piRNA-mediated silencing.

Our screen is relatively blind toward genes that are essential for cellular viability and that therefore could not be evaluated. It is clear that at some point piRNA-specific processes will connect to more general cellular biology. For example, the key heterochromatin protein Su(var)205 (HP1) is likely to be important for piRNA-mediated silencing (Wang and Elgin, 2011), but it could not be evaluated as its knockdown in follicle cells prevented ovarian development.

We identified 144 genes with potential involvement in the somatic piRNA pathway. This set includes (1) true positives, (2) factors with indirect impact on the pathway, and (3) RNAi artifacts. Considering that the *gypsy*-lacZ sensor picks up even moderate defects in TE silencing, the number of factors with indirect impacts will be significant. For example, at least 38 factors can be clearly assigned to aspects of mitochondrial metabolism. As expected for such “indirect” factors, these scored weakly in the screen. We note that the identification of processes or factors that impact the piRNA pathway indirectly might still be valuable. For example, mitochondrial integrity and/or number likely influence TE silencing, as at least two piRNA biogenesis factors are mitochondrial transmembrane proteins. Mitochondrial function is diminished upon aging. As aging has been linked to increased mobility of TEs, it is tempting to speculate that aspects of this might be linked to modulations of piRNA pathway activity.

False positives due to screening artifacts are common for transgenic RNAi screens. They stem from off-target effects of generated siRNAs but can also arise from gain-of-function conditions of genes located in the vicinity of the VDRC transgene insertion. Both aspects, however, are expected to impact our screen only marginally. First, the set of core factors acting in the pathway is unlikely to exceed 30–40 genes, making off-target effects less likely. Second, the screen was based on manual evaluation of a transgenic reporter assay, a highly specific phenotype. And third, only very few genes (if any) will impair TE silencing if overexpressed. These arguments are strongly supported by an experimental validation of nearly all of the 30 tested candidates by an independent RNAi line.

Taken together, our screen represents a highly enriched set of somatic piRNA pathway genes. We speculate that ∼30 factors are either core factors of the somatic piRNA pathway or have direct impact on its function. This testifies to the remarkable complexity of the piRNA pathway compared to the siRNA or miRNA pathways.

### Differences and Commonalities between Somatic and Germline piRNA Pathways

At least two key differences distinguish the piRNA pathways in somatic or germline cells of the *Drosophila* ovary. First, expression of Aub and AGO3 as well as the process of secondary piRNA biogenesis is germline specific (Li et al., 2009; Malone et al., 2009). Second, only germline piRNA clusters are transcribed in both directions. Most of the germline-specific piRNA pathway factors listed in Figure 3E are involved in either of these two aspects. These are Aub/AGO3 and ping-pong factors such as Spindle-E or Vasa on the one side and Rhino and Cutoff on the other side (Klattenhoff et al., 2009; Li et al., 2009; Malone et al., 2009; Pane et al., 2011).

Primary piRNA biogenesis and Piwi-mediated transcriptional silencing are much more likely to be similar between the two cell types, consistent with the literature (Handler et al., 2011; Olivieri et al., 2012; Preall et al., 2012; Sienski et al., 2012; Wang and Elgin, 2011; Zamparini et al., 2011). Several of the newly identified “common factors” acting in both cell types are likely to classify into one of these two categories, including Gasz (primary biogenesis) or CG9754 (silencing).

Considering this, it was surprising to find numerous genes with strong involvement in the somatic pathway that were seemingly dispensable for TE silencing in the germline. Many of these “soma-specific” factors can be grouped into three major processes: nuclear RNA export, the nuclear pore complex, and transcriptional elongation. We believe that these processes are either carried out by related factors in germline cells (e.g., Nxf2 and Nxf3 are germline-specific RNA export factors) or that they are indeed required specifically in somatic cells.

All in all, the identified somatic piRNA pathway factors and the established genetic and cell-biological tools will advance investigations on the *Drosophila* piRNA pathway. Given that several of the key processes and involved factors are conserved in vertebrates, our data will also influence studies in the model systems zebrafish and mouse.

## Experimental Procedures

### *Drosophila* Stocks

Fly stocks are listed in the Supplemental Information.

### RT-qPCR Analysis

Primer sequences and details are given in the Supplemental Information.

### shRNA Transgenes

shRNA lines were cloned into the Valium-20 vector (Ni et al., 2011) modified with a white selection marker and integrated into the attp2 landing site reported in Groth et al., 2004. Hairpin sequences are listed in the Supplemental Information.

### Small RNA Cloning

Small RNA cloning and sequencing was performed as in Jayaprakash et al., 2011. Details are given in the Supplemental Information.

### Antibodies

α-Piwi, α-Aub, and α-AGO3 (rabbit) were described in Brennecke et al., 2007; α-Armi, α-Vret, and α-Yb (rabbit) were described in Handler et al., 2011; α-Piwi and α-Armi (mouse) were described in Saito et al., 2010.

### Immunocytochemistry

IF staining of ovaries and OSCs was done as in Olivieri et al., 2010. All primary antibodies were used at 1:500.

### Northern Blot

Total RNA was isolated from respective knockdowns and separated on a 15% Urea-PAA gel. After transfer onto a membrane, radioactively labeled probes were hybridized over night. Probe sequences and details are given in the Supplemental Information.

### Cell Culture

OSCs were cultured as described as in Niki et al., 2006 and transfected with Cell Line Nucleofector Kit V (Amaxa Biosystems; program T-029). Details are given in the Supplemental Information.

## Accession Numbers

All Illumina data sets (Table S4) were deposited at GEO (GSE45894).

## Supplemental Information

Document S1. Figures S1–S5, Tables S4–S9, and Supplemental Experimental Procedures

Table S1. Primary and Verified Results of the Screen

Table S2. Results of Somatic Quantitative PCR Tests of Positively Scoring Genes

Table S3. Results of Germline Quantitative PCR Tests of Positively Scoring Genes
