# Supplementary material for: The Genetic Makeup of the Drosophila piRNA Pathway
Source: Mol Cell. 2013 Jun 6;50(5):762–77. doi: 10.1016/j.molcel.2013.04.031 (PMC3679447; doi:10.1016/j.molcel.2013.04.031)
Supplement: Document S1. Figures S1–S5, Tables S4–S9, and Supplemental Experimental Procedures [file mmc1.pdf]

Molecular Cell, Volume *50*

Supplemental Information

The Genetic Makeup of the *Drosophila* piRNA Pathway

Dominik Handler, Katharina Meixner, Manfred Pizka, Kathrin Lauss, Christopher Schmied, Franz Sebastian Gruber, and Julius Brennecke

Figure S1.

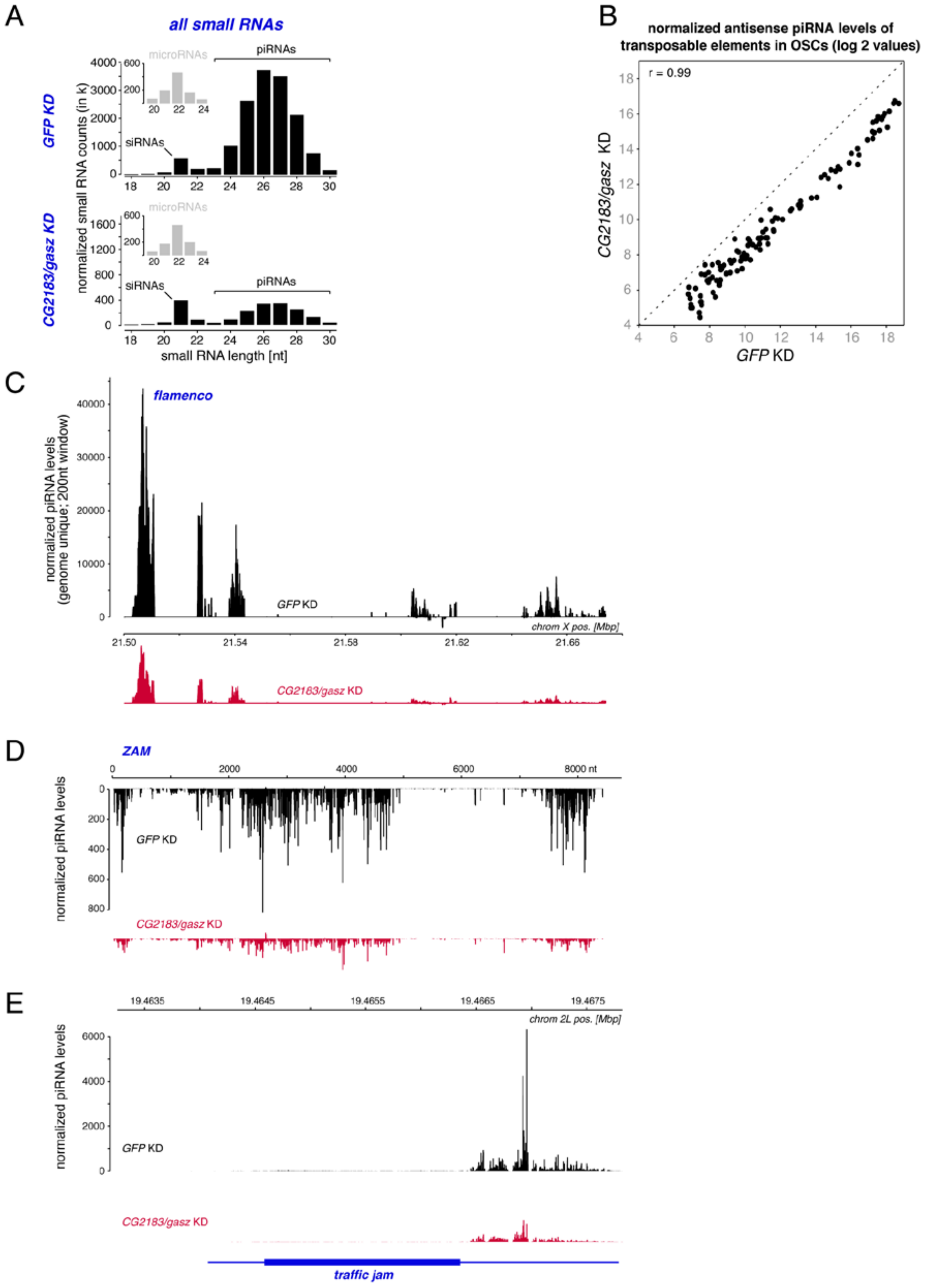

A
all small RNAs
GFP KD
CG2183/gasz KD
normalized small RNA counts (in k)
microRNAs
piRNAs
siRNAs
small RNA length [nt]
B
normalized antisense piRNA levels of transposable elements in OSCs (log 2 values)
r = 0.99
CG2183/gasz KD
GFP KD
C
flamenco
normalized piRNA levels (genome unique; 200nt window)
GFP KD
chrom X pos. [Mbp]
CG2183/gasz KD
D
ZAM
normalized piRNA levels
GFP KD
CG2183/gasz KD
E
chrom 2L pos. [Mbp]
normalized piRNA levels
GFP KD
CG2183/gasz KD
traffic jam

Figure S1. (relates to main Figure 5)

(A) Shown are length profiles of small RNAs (normalized to total miRNA counts) from OSCs transfected with siRNAs against *GFP* (upper panel) or *CG2183/gasz* (lower panel). Small RNAs were split into miRNAs (small insets) and remaining RNAs (siRNAs and piRNAs).

(B) Scatter plot showing log2 values of normalized antisense piRNA levels isolated from OSCs transfected with *GFP* or *CG2183/gasz* siRNAs mapping to annotated TEs in OSCs.

(C) Shown are normalized profiles of genome unique piRNAs (sense up; antisense down) from *GFP* or *CG2183/gasz* siRNA knockdowns in OSCs mapping to the *flamenco* cluster.

(D, E) Shown are normalized profiles of piRNAs (sense up; antisense down) from *GFP* or *CG2183/gasz* siRNA knockdowns in OSCs mapping to the retro-element *ZAM* **(D)** or the traffic jam (tj) locus **(E)**.

Figure S2.

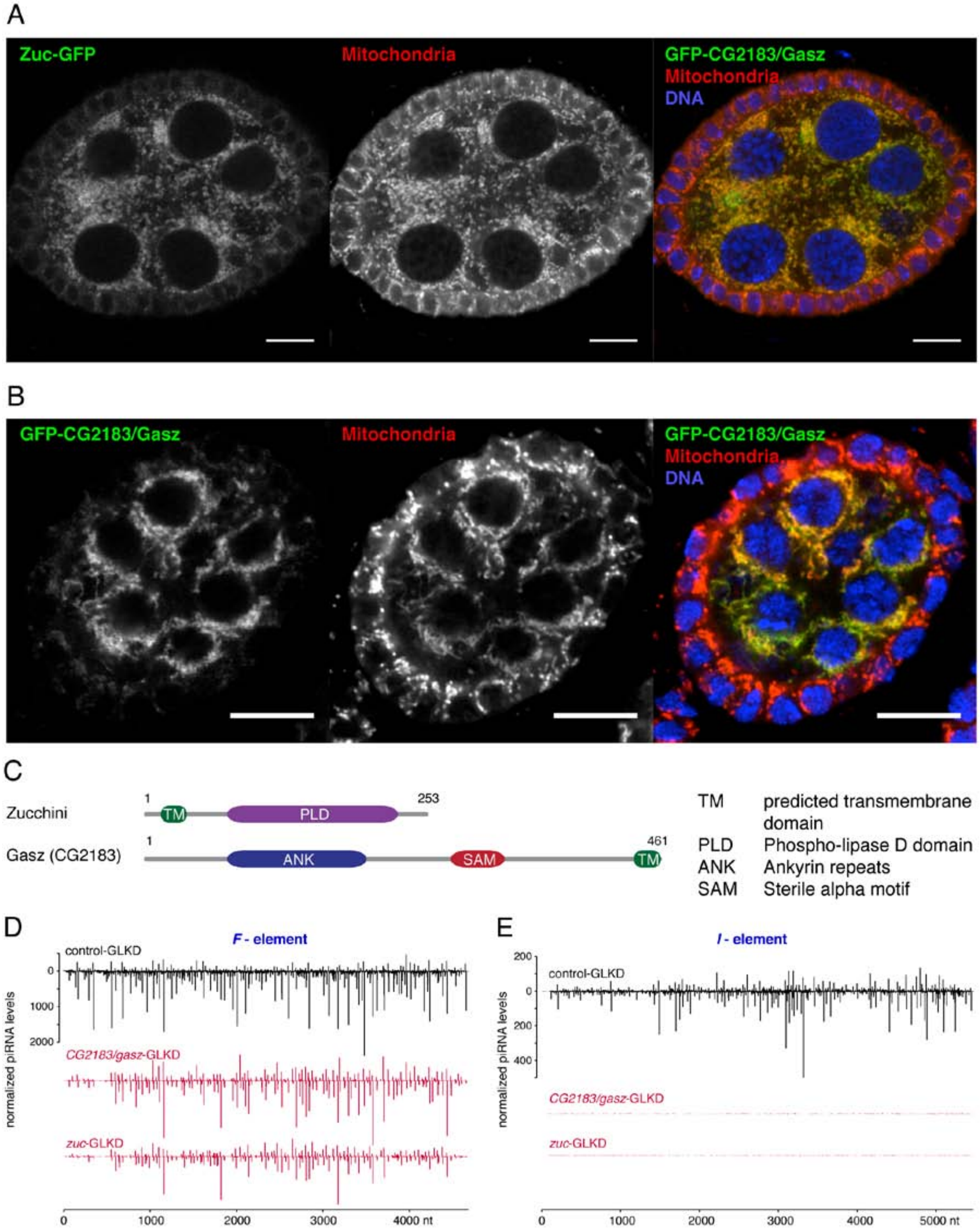
A
Zuc-GFP
Mitochondria
GFP-CG2183/Gasz
Mitochondria
DNA
B
GFP-CG2183/Gasz
Mitochondria
GFP-CG2183/Gasz
Mitochondria
DNA
C
Zucchini
1
TM
PLD
253
Gasz (CG2183)
1
ANK
SAM
461
TM
TM predicted transmembrane domain
PLD Phospho-lipase D domain
ANK Ankyrin repeats
SAM Sterile alpha motif
D
F - element
control-GLKD
0
1000
2000
normalized piRNA levels
CG2183/gasz-GLKD
zuc-GLKD
0
1000
2000
3000
4000 nt
E
I - element
200
0
200
400
control-GLKD
normalized piRNA levels
CG2183/gasz-GLKD
zuc-GLKD
0
1000
2000
3000
4000
5000 nt

Figure S2. (relates to main Figure 6)

(A, B) Confocal sections of egg chambers expressing GFP tagged Zucchini **(A)** or GFP tagged CG2183/Gasz **(B)** stained for mitochondria (red) and DNA (blue).

(C) Cartoon depicting the predicted domain architecture of Zucchini and CG2183/Gasz. Shown are the full-length protein sequences annotated with their protein domains. Transmembrane helices were predicted with TMHMM.

(D) Cartoon depicting the predicted domain architecture of Zucchini and CG2183/Gasz. Shown are the full-length protein sequences annotated with their protein domains. Transmembrane helices were predicted with TMHMM.

(E-F) Shown are normalized piRNA profiles (sense up; antisense down) from ovaries with indicated germline knockdowns mapping to the *F*-element **(E)** or to the *I*-element **(F).**

Figure S3.

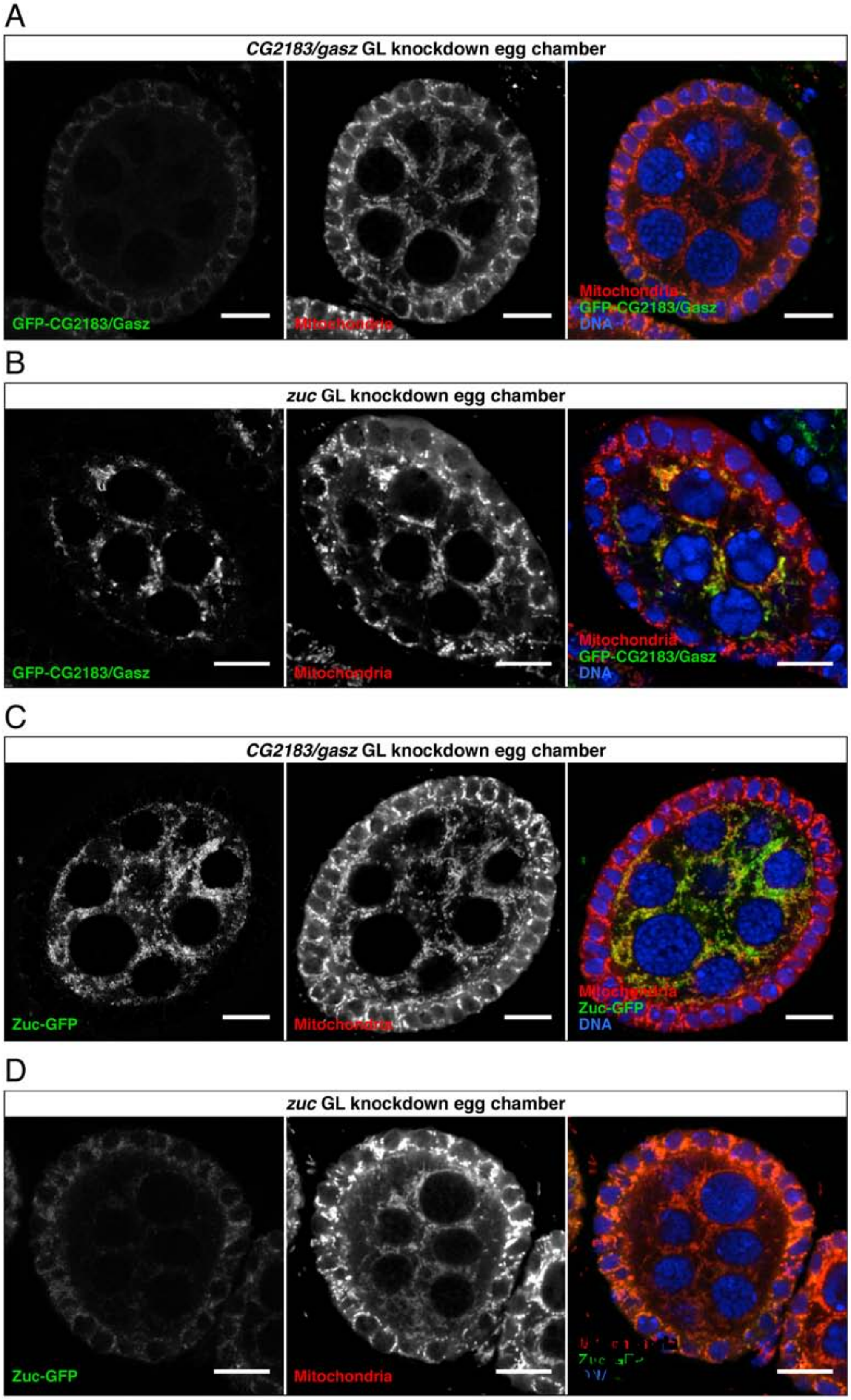
A
CG2183/gasz GL knockdown egg chamber
GFP-CG2183/Gasz
Mitochondria
Mitochondria
GFP-CG2183/Gasz
DNA
B
zuc GL knockdown egg chamber
GFP-CG2183/Gasz
Mitochondria
Mitochondria
GFP-CG2183/Gasz
DNA
C
CG2183/gasz GL knockdown egg chamber
Zuc-GFP
Mitochondria
Mitochondria
Zuc-GFP
DNA
D
zuc GL knockdown egg chamber
Zuc-GFP
Mitochondria

Figure S3. (relates to main Figure 7)

(A, B) Confocal sections of egg chambers expressing GFP-CG2183/Gasz stained for mitochondria (red) and DNA (blue). Shown are germline specific knockdowns for *CG2183/gasz* **(A)** or *zuc* **(B)**.

(C, D) Confocal sections of egg chambers expressing Zuc-GFP stained for mitochondria (red) and DNA (blue). Shown are germline specific knockdowns for *CG2183/gasz* **(C)** or *zuc* **(D)**.

Figure S4.

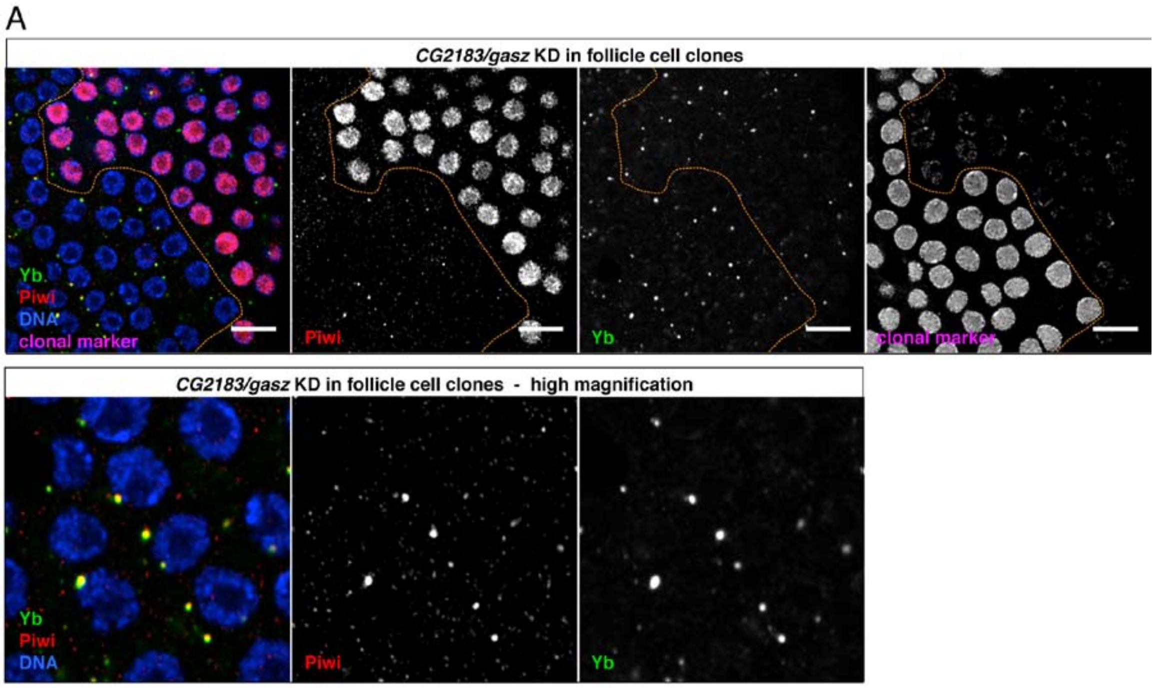

Figure S4. (relates to main Figure 7)

(A) The upper panels show a confocal section through the follicular epithelium of an egg chamber stained for Yb (green), Piwi (red) and DNA (blue). Knockdown of *CG2183/gasz* has been clonally induced (clonal marker in magenta; outlined by dashed line). The lower panels represent high magnification images confirming co-localization of Piwi and Yb.

Figure S5.

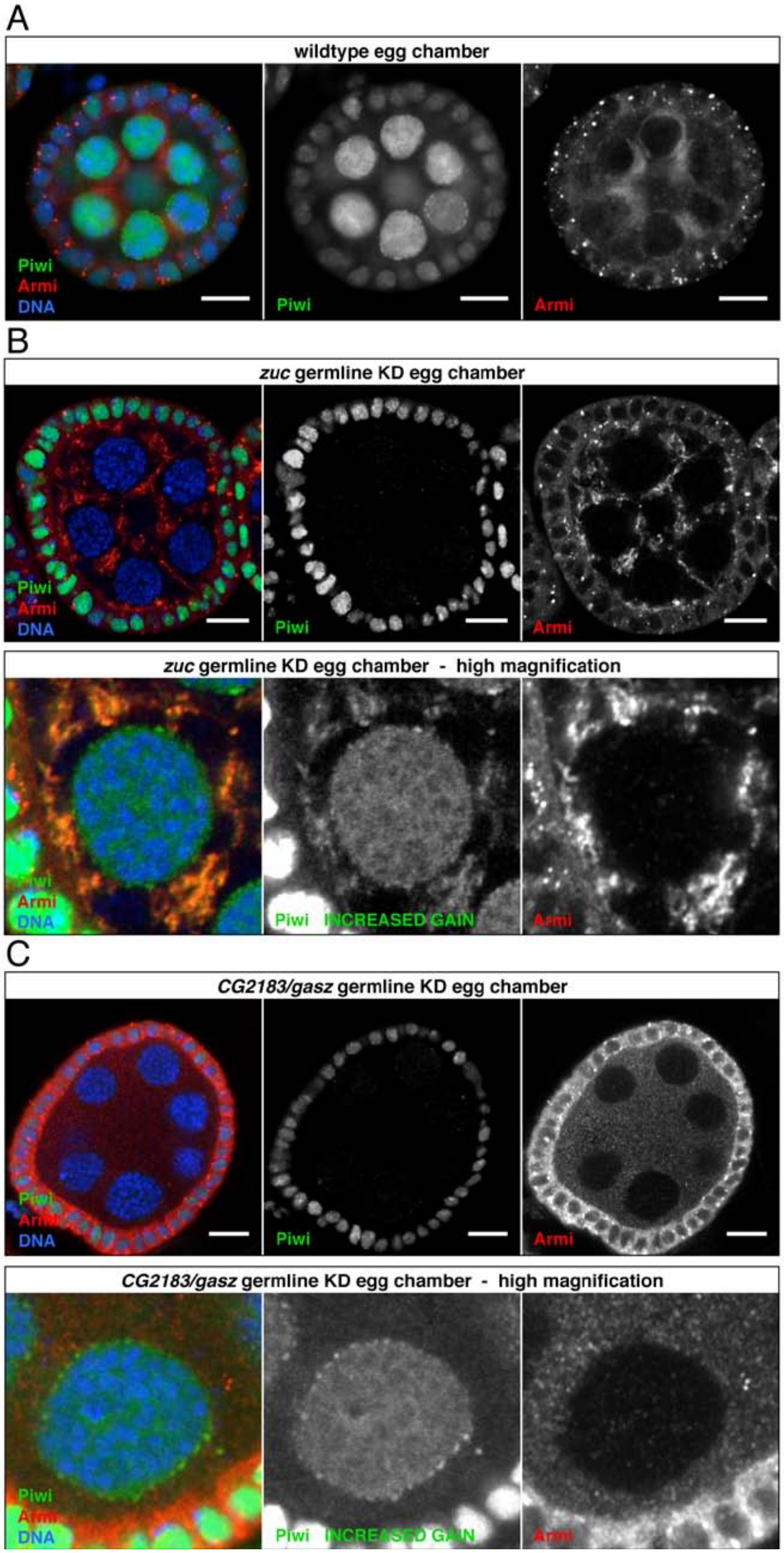
A
wildtype egg chamber
Piwi
Armi
DNA
Piwi
Armi
B
zuc germline KD egg chamber
Piwi
Armi
DNA
Piwi
Armi
zuc germline KD egg chamber - high magnification
Piwi
Armi
DNA
Piwi INCREASED GAIN
Armi
C
CG2183/gasz germline KD egg chamber
Piwi
Armi
DNA
Piwi
Armi
CG2183/gasz germline KD egg chamber - high magnification
Piwi
Armi
DNA
Piwi INCREASED GAIN
Armi

Figure S5. (relates to main Figure 7)

(A-C) Confocal sections of egg chambers expressing GFP-Piwi and stained for Armi (red) and DNA (blue). Shown are egg chambers from wildtype flies **(A)**, germline specific *zuc* knockdown **(B)** or germline specific *CG2183/gasz* knockdown **(C)**. For panels **(B)** and **(C)** high magnification images with increased gain for the Piwi channel are shown in addition.

Table S4. Illumina sequencing data sets used in this study

Origin	Method	Genotype	GEO
**Flies**	small RNA-seq	*UAS-Dcr-2*; *NGT*; *nosGAL4*, *Burdock*-lacZ x *w[1118]*	GSE45894
		UAS-Dcr-2; *NGT*; *nosGAL4*, *Burdock*-lacZ x VDRC-13762	GSE45894
		MTD x shRNA-*armi*	GSE38728
		MTD x shRNA-*zuc*	GSE38728
		MTD x *w[1118]*	GSE38728
**OSC**	RNA-seq	Wildtype (replicate #1)	GSE45894
		Wildtype (replicate #2)	GSE45894
	small RNA-seq	si-GFP KD	GSE45894
		si-*CG2183/gasz* KD	GSE45894

Table S5. Used fly stocks

MTD-GAL4 (Ni et al., 2011)
y,w,hsFlp122;;act<CD2>GAL4, UAS-GFP (Olivieri et al., 2010)
Short hairpin RNA (shRNA) lines were cloned into the Valium-20 or the Valium-22 vector (Ni et al, 2011) modified with a white selection marker and integrated into the attp2 landing site (Markstein et al., 2008). Hairpin sequences, see Table S5
armi shRNA, *SoYb* shRNA, *BoYb* shRNA, (Handler et al., 2011)
zuc shRNA (TRiP # GL00111);
eGFP_CG2183/Gasz and eGFP_CG9754 were cloned by inserting N-terminal eGFP via bacterial recombineering into genomic rescue constructs and integrated into the attp2 landing site.
eGFP_Zuc was cloned by inserting C-terminal eGFP via bacterial recombineering into a genomic rescue construct that was integrated into the attp2 landing site.
flam$^{(R)}$*;;gypsy-lacZ* (Sarot et al., 2004)
flam$^{(P)}$*;;gypsy-lacZ* (Sarot et al., 2004)
tj-GAL4, gypsy-lacZ/Cyo;
UAS-Dcr-2; *NGT*; *nosGAL4, Burdock*-lacZ/Tm3,Ser
All flies have been aged 5-7 days at 25°C before analysis

Table S6. Primer for QPCR analysis

Gene	Sequence	
rp49	fw:	CCGCTTCAAGGGACAGTATCTG
	rv:	ATCTCGCCGCAGTAAACGC
lacZ	fw:	AATGTTGATGAAAGCTGGCTAC
	rv:	GCTCAGGTCAAATTCAGACG
zam	fw:	ACTTGACCTGGATACACTCACAAC
	rv:	GAGTATTACGGCGACTAGGGATAC
gypsy	fw:	CAACAATCTGAACCCACCAATCT
	rv:	TATGAACATCATGAGGGTGAACG
HeT-A	fw:	CGCGCGGAACCCATCTTCAGA
	rv:	CGCCGCAGTCGTTTGGTGAGT
Burdock	fw:	CGGTAAAATCGCTTCATGGT
	rv:	ACGTTGCATTTCCCTGTTTC
Act5c	fw:	AAGTTGCTGCTCTGGTTGTCG
	rv:	GCCACACGCAGCTCATTGTAG
mdg1	fw:	AAACTCCAACTCCCAATCcG
	rv:	AGTGGTCCCTCGCAGTCGTT
blood	fw:	AACAATAGAAAGAAGCCACCGAAC
	rv:	AGTCATGGACTATTGAGGGTGTTG

Table S7. Oligos used for cloning of shRNA constructs

Gene	Sequence
	**Cloned into W20**
CG9754	fw: ctagcagtAACGGCTACGCTGTACAAGAAtagttatattcaagcataTTCTTGTACAGCGTAGCCGTTgcg<br>rv: aattcgcAACGGCTACGCTGTACAAGAAtatgcttgaatataactaTTCTTGTACAGCGTAGCCGTTactg
Acn	fw: ctagcagtACCGCCTATCAGACTACTAGAtagttatattcaagcataTCTAGTAGTCTGATAGGCGGTgcg<br>rv: aattcgcACCGCCTATCAGACTACTAGAtatgcttgaatataactaTCTAGTAGTCTGATAGGCGGTactg
TfIIS	fw: ctagcagtCACGGATATGAAGTACAAGAAtagttatattcaagcataTTCTTGTACTTCATATCCGTGgcg<br>rv: aattcgcCACGGATATGAAGTACAAGAAtatgcttgaatataactaTTCTTGTACTTCATATCCGTGactg
CG3893	fw: ctagcagtGCCGTGTGATCTACAAAGACAtagttatattcaagcataTGTCTTTGTAGATCACACGGCgcg<br>rv: aattcgcGCCGTGTGATCTACAAAGACAtatgcttgaatataactaTGTCTTTGTAGATCACACGGCactg
Nup58	fw: ctagcagtACCACAAGGAGCACTACCTAAtagttatattcaagcataTTAGGTAGTGCTCCTTGTGGTgcg<br>rv: aattcgcACCACAAGGAGCACTACCTAAtatgcttgaatataactaTTAGGTAGTGCTCCTTGTGGTactg
CG2183	fw: ctagcagtTCCCTTGTTCATTGACTTCAAtagttatattcaagcataTTGAAGTCAATGAACAAGGGAgcg<br>rv: aattcgcTCCCTTGTTCATTGACTTCAAtatgcttgaatataactaTTGAAGTCAATGAACAAGGGAactg
sbr	fw: ctagcagtAAGCGATGCTCTCCATATCAAtagttatattcaagcataTTGATATGGAGAGCATCGCTTgcg<br>rv: aattcgcAAGCGATGCTCTCCATATCAAtatgcttgaatataactaTTGATATGGAGAGCATCGCTTactg
Atu	fw: ctagcagtCCGCTCGTAGTAAGAGATCAAtagttatattcaagcataTTGATCTCTTACTACGAGCGGgcg<br>rv: aattcgcCCGCTCGTAGTAAGAGATCAAtatgcttgaatataactaTTGATCTCTTACTACGAGCGGactg
Nup54	fw: ctagcagtAGCGAAGATACTTGAACATAAtagttatattcaagcataTTATGTTCAAGTATCTTCGCTgcg<br>rv: aattcgcAGCGAAGATACTTGAACATAAtatgcttgaatataactaTTATGTTCAAGTATCTTCGCTactg
asf1	fw: ctagcagtCCGCGTGGGCTACTATGTGAAtagttatattcaagcataTTCACATAGTAGCCCACGCGGgcg<br>rv: aattcgcCCGCGTGGGCTACTATGTGAAtatgcttgaatataactaTTCACATAGTAGCCCACGCGGactg
CG9915	fw: ctagcagtGTGGTTCTAGACGTTCTAGAAtagttatattcaagcataTTCTAGAACGTCTAGAACCACgcg<br>rv: aattcgcGTGGTTCTAGACGTTCTAGAAtatgcttgaatataactaTTCTAGAACGTCTAGAACCACactg
Patr-1	fw: ctagcagtCACGCCGTATATCGACGACTAtagttatattcaagcataTAGTCGTCGATATACGGCGTGgcg<br>rv: aattcgcCACGCCGTATATCGACGACTAtatgcttgaatataactaTAGTCGTCGATATACGGCGTGactg
CG40228	fw: ctagcagtCACCTCCGAAGAGAAAGAATAtagttatattcaagcataTATTCTTTCTCTTCGGAGGTGgcg<br>rv: aattcgcCACCTCCGAAGAGAAAGAATAtatgcttgaatataactaTATTCTTTCTCTTCGGAGGTGactg
CG3689	fw: ctagcagtACCAACGATAGCGCTACCTAAtagttatattcaagcataTTAGGTAGCGCTATCGTTGGTgcg<br>rv: aattcgcACCAACGATAGCGCTACCTAAtatgcttgaatataactaTTAGGTAGCGCTATCGTTGGTactg
mago	fw: ctagcagtCACCACCTCAAGCATAGCCTAtagttatattcaagcataTAGGCTATGCTTGAGGTGGTGgcg<br>rv: aattcgcCACCACCTCAAGCATAGCCTAtatgcttgaatataactaTAGGCTATGCTTGAGGTGGTGactg
RnpS1	fw: ctagcagtACCCCAGTTCAATAGGTTCAAtagttatattcaagcataTTGAACCTATTGAACTGGGGTgcg<br>rv: aattcgcACCCCAGTTCAATAGGTTCAAtatgcttgaatataactaTTGAACCTATTGAACTGGGGTactg
gw	fw: ctagcagtTAGCGCTTCTAACTTAACTAAtagttatattcaagcataTTAGTTAAGTTAGAAGCGCTAgcg<br>rv: aattcgcTAGCGCTTCTAACTTAACTAAtatgcttgaatataactaTTAGTTAAGTTAGAAGCGCTAactg
Nxt1	fw: ctagcagtCTCCGTCAAGTTCGCAGATCAtagttatattcaagcataTGATCTGCGAACTTGACGGAGgcg<br>rv: aattcgcCTCCGTCAAGTTCGCAGATCAtatgcttgaatataactaTGATCTGCGAACTTGACGGAGactg
ball	fw: ctagcagtACGGCGGTTGATTCACGTACAtagttatattcaagcataTGTACGTGAATCAACCGCCGTgcg<br>rv: aattcgcACGGCGGTTGATTCACGTACAtatgcttgaatataactaTGTACGTGAATCAACCGCCGTactg
pcm	fw: ctagcagtACCGACGAACGAGACTATAGAtagttatattcaagcataTCTATAGTCTCGTTCGTCGGTgcg<br>rv: aattcgcACCGACGAACGAGACTATAGAtatgcttgaatataactaTCTATAGTCTCGTTCGTCGGTactg
CG8211	fw: ctagcagtCTGGTGCTCAACTATTCGATAtagttatattcaagcataTATCGAATAGTTGAGCACCAGgcg<br>rv: aattcgcCTGGTGCTCAACTATTCGATAtatgcttgaatataactaTATCGAATAGTTGAGCACCAGactg
Actr13E	fw: ctagcagtGCGACGCGAAGTCACAGTTGAtagttatattcaagcataTCAACTGTGACTTCGCGTCGCgcg<br>rv: aattcgcGCGACGCGAAGTCACAGTTGAtatgcttgaatataactaTCAACTGTGACTTCGCGTCGCactg
veli	fw: ctagcagtCACGAGAAGGCCGTAGAGCTAtagttatattcaagcataTAGCTCTACGGCCTTCTCGTGgcg<br>rv: aattcgcCACGAGAAGGCCGTAGAGCTAtatgcttgaatataactaTAGCTCTACGGCCTTCTCGTGactg
SRPK	fw: ctagcagtCACCTTGAAAGGACAATCAAAtagttatattcaagcataTTTGATTGTCCTTTCAAGGTGgcg<br>rv: aattcgcCACCTTGAAAGGACAATCAAAtatgcttgaatataactaTTTGATTGTCCTTTCAAGGTGactg
CG5859	fw: ctagcagtCCGCAATGCGTTGCTATGTAAtagttatattcaagcataTTACATAGCAACGCATTGCGGgcg<br>rv: aattcgcCCGCAATGCGTTGCTATGTAAtatgcttgaatataactaTTACATAGCAACGCATTGCGGactg
SelD	fw: ctagcagtCAGTACCAAGATGACAGAGAAtagttatattcaagcataTTCTCTGTCATCTTGGTACTGgcg<br>rv: aattcgcCAGTACCAAGATGACAGAGAAtatgcttgaatataactaTTCTCTGTCATCTTGGTACTGactg
CG6020	fw: ctagcagtCAGGGACTTCGAGACCAAGAAtagttatattcaagcataTTCTTGGTCTCGAAGTCCCTGgcg<br>rv: aattcgcCAGGGACTTCGAGACCAAGAAtatgcttgaatataactaTTCTTGGTCTCGAAGTCCCTGactg
TSG101	fw: ctagcagtCCGAGTTCAGGAGAAAGTTAAtagttatattcaagcataTTAACTTTCTCCTGAACTCGGgcg<br>rv: aattcgcCCGAGTTCAGGAGAAAGTTAAtatgcttgaatataactaTTAACTTTCTCCTGAACTCGGactg

spi	fw: ctagcagtCAAGGAGATCGACAATACTTAtagttatattcaagcataTAAGTATTGTCGATCTCCTTGgcg<br>rv: aattcgcCAAGGAGATCGACAATACTTAtatgcttgaatataactaTAAGTATTGTCGATCTCCTTGactg
mr	fw: ctagcagtATCGATGGTTGTAGACATATAtagttatattcaagcataTATATGTCTACAACCATCGATgcg<br>rv: aattcgcATCGATGGTTGTAGACATATAtatgcttgaatataactaTATATGTCTACAACCATCGATactg
	**Cloned into W22**
armi	fw: ctagcagtTTCGCTGTCAAGCTAAGTTTAtagttatattcaagcataTAAACTTAGCTTGACAGCGAAgcg<br>rv: aattcgcTTCGCTGTCAAGCTAAGTTTAtatgcttgaatataactaTAAACTTAGCTTGACAGCGAAactg
krimp	fw: ctagcagtCAGATTGGGAGACTACGAATAtagttatattcaagcataTATTCGTAGTCTCCCAATCTGgcg<br>rv: aattcgcCAGATTGGGAGACTACGAATAtatgcttgaatataactaTATTCGTAGTCTCCCAATCTGactg
AGO3	fw: ctagcagtTTGGTTGGAAATATTGTCTTAtagttatattcaagcataTAAGACAATATTTCCAACCAAgcg<br>rv: aattcgcTTGGTTGGAAATATTGTCTTAtatgcttgaatataactaTAAGACAATATTTCCAACCAAactg
SoYb	fw: ctagcagtAAGGTTCAAAGTATCAGCGAAtagttatattcaagcataTTCGCTGA TACTTTGAACCTTgcg<br>rv: aattcgcAAGGTTCAAAGTATCAGCGAAtatgcttgaatataactaTTCGCTGATACTTTGAACCTTactg
BoYb	fw: aattcgcCAGCATCAGTTGTGCGATCAAtatgcttgaatataactaTTGATCGCACAACTGATGCTGactg<br>rv: ctagcagtCAGCATCAGTTGTGCGATCAAtagttatattcaagcataTTGATCGCACAACTGATGCTGgcg
	**Other**
zuc	TRiP.GL0111 / Bloomingtion stock: 35227

Table S8. Probes used for Northern Blots

**Name**	**Sequence**
Idefix	AAACTACTGGCAATCGTTTGGGAA
miR-310	AAAGGCCGGGAAGTGTGCAATA

Table S9. siRNAs used for RNAi in OSCs

**Gene**	**sequence**	
GFP	Guide:<br>Passenger:	ACUUCAGGGUCAGCUUGCCTT<br>GGCAAGCUGACCCUGAAGUTT
armi	Guide:<br>Passenger:	UAAACUUAGCUUGACAGCGTT<br>CGCUGUCAAGCUAAGUUUATT
CG9754	Guide:<br>Passenger:	UUCUUGUACAGCGUAGCCGUU<br>CGGCUACGCUGUACAAGAAUU
CG2183/gasz	Guide:<br>Passenger:	UUGAAGUCAAUGAACAAGGGAUU<br>CCUUGUUCAUUGACUUCAACGUU
zuc	Guide:<br>Passenger:	UUGUUGUGCAUCAAGUUCGTT<br>CGAACUUGAUGCACAACAATT

SUPPLEMENTAL EXPERIMENTAL PROCEDURES

X-Gal staining

Ovaries from 5-7 day old flies were dissected into ice cold PBS (max 30 min), fixed in 0.5% Glutaraldehyde/PBS (RT, 15 min), and washed with PBS. The staining reaction was performed with staining solution (10mM PBS, 1mM MgCl2, 150 mM NaCl, 3 mM potassium ferricyanide, 3 mM potassium ferrocyanide, 0.1% Triton, 0.1% X-Gal) at room temperature over night (*gypsy* reporter) or for 2 hours (*Burdock* reporter).

Cell culture

Act>GFP-CG9754, Act>GFP-CG2183/Gasz and Act>Zuc-GFP have been constructed by Gateway cloning using full cDNA amplicons of the genes.

Act>Zuc ΔN-GFP and Act>GFP-CG2183/Gasz ΔC have been constructed by gateway cloning. Sequences used for cloning were *zuc* cDNA (bp 116-759) and *CG2183*/*gasz* cDNA (bp 4-1305)

Transposon QPCR analysis

cDNA was prepared via random priming of 1µg total RNA isolated from ovaries of 5-7 day old flies. Quantitative PCR was performed using a homemade QPCR master mix (20 mM Tris pH 8.3, 100 mM KCl, 5 mM MgCl2, 0.5 mM each dNTP. 1x Evagreen, 40 µL/ml TAQ).

Each experiment was performed in biological triplicates with technical duplicates. Relative RNA levels were calculated by the 2-ΔΔCT method (Livak and Schmittgen, 2001) and normalized to rp49 levels. Fold enrichments were calculated in comparison to respective RNA levels obtained from heterozygous flies, from flies not harboring a

knockdown hairpin or from control siRNA transfections into OSCs.

Northern Blot analysis

Total RNA was isolated from respective knockdowns and separated on a 15% polyacrylamide urea gel. RNA was transferred to Amersham Hybond-NX (RPN303T) membrane and crosslinked by EDC (1-ethyl-3-(3- dimethylaminoprophy) carbodiimide) for 1 hour (Pall and Hamilton, 2008). The membrane was pre-hybridized in Church Buffer and hybridized to probes overnight at 37°C. The membrane was washed 3 times 10 minutes with 2xSSC, 0.1% SDS and exposed.

small RNA cloning

Small RNA cloning and sequencing was performed as described (Jayaprakash et al., 2011). In brief, 20 µg of total RNA was isolated from ovaries or OSCs by TRIzol and Phenol/Chloroform extraction, was resolved on a denaturing polyacrylamide gel and RNAs corresponding to 18-28 nt were isolated and subjected to ligations of 3'-, and 5'-adaptors followed by reverse transcription and PCR amplification; libraries were sequenced on GAII or HiSeq2000 platforms (Illumina).

The RNA cloning strategy introduces 4 random nucleotides at 3' end of the 5' linker and 5' end of the 3' linker, which reduces ligation biases (Jayaprakash et al., 2011). Reads were first stripped of the 3' adaptor and then the introduced 4 random nucleotides at each end of the read were removed.

Reads were mapped to the genome (100% match; release 5). For piRNA cluster mapping we considered genome-unique mappers, for TE mappings (Repbase; (Jurka et al., 2005) all mappers (up to 3 MM) have been considered. Libraries were normalized to 1 Mio

miRNA reads. Small RNAs mapping to rRNAs, tRNAs and snoRNAs were excluded. The calculation of TE piRNA levels was based on antisense piRNAs. Ping-pong signatures were calculated as previously described (Malone et al., 2009).

RNA sequencing (RNA-seq)

mRNA from wildtype OSCs was selected with Dynabeads Oligo(dT) (Invitrogen) from total RNA, fragmented and reverse transcribed with random hexamers. Strand-specific libraries were prepared using the UDG-digestion-based strategy, cloned with NEBNext ChIP-Seq Library Prep Reagent Set for Illumina (NEB) and sequenced on a Genome Analyzer II (Illumina).

This yielded ~6–20 million genome- and transcriptome-mappable reads. For the computational analyses, we first extracted high quality bases from every read (6-36 nt) and mapped these to the *Drosophila* genome as well as to the FlyBase transcriptome. Uniquely aligned reads were used for quantification of gene expression levels according to coordinates in the Flybase gene annotation (r5.38) by calculating RPKM values.

Gene expression enrichment analysis

Expression data was obtained form FlyAtlas (Chintapalli et al., 2007). For genes tested multiple times an average value was calculated. Ratios between expression values of individual tissues and the value for “whole fly” have been calculated for all genes. Tissue enrichment analysis was performed using Wilcoxon Rank Sum and Signed Rank Test implementation in R. The lists used for statistical analysis contained all tested genes (background set) or all genes scoring positively in the screen.

KEGG tem analysis

KEGG analysis was done with DAVID (Huang et al., 2009b; 2009a) online tool. All tested genes have been used as background list, whereas genes showing a 'no ovary' phenotype were used for analysis. Presented P-values have been corrected for multiple testing using the Benjamini-Hochberg method.

GO term analysis

GO analysis was performed using GOrilla (Eden et al., 2007; 2009) online tool using two ranked lists of genes. As target set all positively scoring or all positively scoring genes depleted for mitochondrial genes have been used. All genes tested in the screen have been used as background list. Presented P-values have been corrected for multiple testing using the Benjamini-Hochberg method.

Statistical analyses

We used statistical packages implemented in R 2.15.0 for all calculations and plots in this study. For data visualization in box plot format we used standard features of ggplot2 boxplot function: horizontal bar represents median, the box depicts 25th and 75th percentile (lower and upper quartile respectively), whiskers represent sample minimum (lower) and maximum (upper); outliers are shown as circles. Statistical significances in Fig. 4D and Fig. 7D were calculated with Wilcoxon Rank Sum and the Signed Rank Test implementation in R.

Image quantification

The images were copied to a 50% downsampled map where the initial segmentation was performed. The Armitage and DAPI channels were added together to get a good representation of the whole cell and a multiresolution segmentation was used to define object borders. By evaluating intensity and standard deviation those objects belonging to cells were defined and classified. Objects of the cell class were merged, the borders refined by growing using surface tension and then the objects were synchronized back onto the main map. From within the cell class a quantile of pixel intensities of 95% from the Armi channel was calculated and used as a threshold to segment the Yb-bodies. The sum of intensities from the Piwi channel within Yb-bodies was calculated and divided by the sum of intensities of the surrounding cytoplasm. This gives a relative measure of how much of the Piwi signal colocalized with Yb-bodies.

SUPPLEMENTAL REFERENCES

Chintapalli, V.R., Wang, J., and Dow, J.A.T. (2007). Using FlyAtlas to identify better Drosophila melanogaster models of human disease. Nat Genet *39*, 715–720.

Eden, E., Lipson, D., Yogev, S., and Yakhini, Z. (2007). Discovering motifs in ranked lists of DNA sequences. PLoS Comput. Biol. *3*, e39.

Eden, E., Navon, R., Steinfeld, I., Lipson, D., and Yakhini, Z. (2009). GOrilla: a tool for discovery and visualization of enriched GO terms in ranked gene lists. BMC Bioinformatics *10*, 48.

Handler, D., Olivieri, D., Novatchkova, M., Gruber, F.S., Meixner, K., Mechtler, K., Stark, A., Sachidanandam, R., and Brennecke, J. (2011). A systematic analysis of Drosophila TUDOR domain-containing proteins identifies Vreteno and the Tdrd12 family as essential primary piRNA pathway factors. The EMBO Journal.

Huang, D.W., Sherman, B.T., and Lempicki, R.A. (2009a). Bioinformatics enrichment tools: paths toward the comprehensive functional analysis of large gene lists. Nucleic Acids Res *37*, 1–13.

Huang, D.W., Sherman, B.T., and Lempicki, R.A. (2009b). Systematic and integrative analysis of large gene lists using DAVID bioinformatics resources. Nat Protoc *4*, 44–57.

Jayaprakash, A.D., Jabado, O., Brown, B.D., and Sachidanandam, R. (2011). Identification and remediation of biases in the activity of RNA ligases in small-RNA deep sequencing. Nucleic Acids Res *39*, e141.

Jurka, J., Kapitonov, V.V., Pavlicek, A., Klonowski, P., Kohany, O., and Walichiewicz, J. (2005). Repbase Update, a database of eukaryotic repetitive elements. Cytogenet Genome Res *110*, 462–467.

Livak, K.J., and Schmittgen, T.D. (2001). Analysis of relative gene expression data using real-time quantitative PCR and the 2(-Delta Delta C(T)) Method. Methods *25*, 402–408.

Malone, C.D., Brennecke, J., Dus, M., Stark, A., Mccombie, W.R., Sachidanandam, R., and Hannon, G.J. (2009). Specialized piRNA pathways act in germline and somatic tissues of the Drosophila ovary. Cell *137*, 522–535.

Markstein, M., Pitsouli, C., Villalta, C., Celniker, S.E., and Perrimon, N. (2008). Exploiting position effects and the gypsy retrovirus insulator to engineer precisely expressed transgenes. Nat Genet *40*, 476–483.

Olivieri, D., Sykora, M.M., Sachidanandam, R., Mechtler, K., and Brennecke, J. (2010). An in vivo RNAi assay identifies major genetic and cellular requirements for primary piRNA biogenesis in Drosophila. The EMBO Journal *29*, 3301–3317.

Pall, G.S., and Hamilton, A.J. (2008). Improved northern blot method for enhanced detection of small RNA. Nat Protoc *3*, 1077–1084.

Sarot, E., Payen-Groschêne, G., Bucheton, A., and Pélisson, A. (2004). Evidence for a piwi-dependent RNA silencing of the gypsy endogenous retrovirus by the Drosophila melanogaster flamenco gene. Genetics *166*, 1313–1321.
